# Hydrogen peroxide-induced oxidative damage and protective role of peroxiredoxin 6 protein via EGFR/ERK signaling pathway in RPE cells

**DOI:** 10.3389/fnagi.2023.1169211

**Published:** 2023-07-17

**Authors:** Xiaodong Chen, Radouil Tzekov, Mingyang Su, Yusheng Zhu, Aidong Han, Wensheng Li

**Affiliations:** ^1^Department of Ophthalmology, Xi’an No. 1 Hospital, Shaanxi Institute of Ophthalmology, First Affiliated Hospital of Northwest University, Northwest University, Xi’an, Shaanxi, China; ^2^Xiamen Eye Center of Xiamen University, Xiamen University, Xiamen, Fujian, China; ^3^Department of Ophthalmology, University of South Florida, Tampa, FL, United States; ^4^State Key Laboratory for Cellular Stress Biology, School of Life Sciences, Xiamen University, Xiangan, Xiamen, China; ^5^Shanghai Aier Eye Hospital, Shanghai, China; ^6^Shanghai Aier Eye Institute, Shanghai, China; ^7^Aier School of Ophthalmology, Central South University, Changsha, Hunan, China

**Keywords:** Peroxiredoxin 6, retinal pigment epithelium cell, oxidative stress, epidermal growth factor receptor, extracellular signal-regulated kinase

## Abstract

**Introduction:**

Damage to retinal pigment epithelium (RPE) cells caused by oxidative stress is closely related to the pathogenesis of several blinding retinal diseases, such as age-related macular degeneration (AMD), retinitis pigmentosa, and other inherited retinal degenerative conditions. However, the mechanisms of this process are poorly understood. Hence, the goal of this study was to investigate hydrogen peroxide (H_2_O_2_)-induced oxidative damage and protective role of peroxiredoxin 6 (PRDX6) protein via EGFR/ERK signaling pathway in RPE cells.

**Methods:**

Cells from a human RPE cell line (ARPE-19 cells) were treated with H_2_O_2_, and then cell viability was assessed using the methyl thiazolyl tetrazolium assay. Cell death and reactive oxygen species (ROS) were detected by flow cytometry. The levels of PRDX6, epidermal growth factor receptor (EGFR), P38 mitogen-activated protein kinase (P38MAPK), c-Jun N-terminal kinase (JNK), and extracellular signal-regulated kinase (ERK) were detected by Western blot assay. PRDX6 and EGFR were also detected via immunofluorescence staining.

**Results:**

Our results show that H_2_O_2_ inhibited cell viability, induced cell death, and increased ROS levels in ARPE-19 cells. It was also found that H_2_O_2_ decreased the levels of PRDX6, EGFR, and phosphorylated ERK but increased the levels of phosphorylated P38MAPK and JNK. PRDX6 overexpression was found to attenuate H_2_O_2_-induced inhibition of cell viability and increased cell death and ROS production in ARPE-19 cells. PRDX6 overexpression also increased the expression of EGFR and alleviated the H_2_O_2_-induced decrease in EGFR and phosphorylated ERK. Moreover, inhibition of epidermal growth factor-induced EGFR and ERK signaling in oxidative stress was partially blocked by PRDX6 overexpression.

**Discussion:**

Our findings indicate that PRDX6 overexpression protects RPE cells from oxidative stress damage caused by decreasing ROS production and partially blocking the inhibition of the EGFR/ERK signaling pathway induced by oxidative stress. Therefore, PRDX6 shows promise as a therapeutic target for the prevention of RPE cell damage caused by oxidative stress associated with retinal diseases.

## 1. Introduction

Age-related macular degeneration (AMD) is the most common blinding retinal disease in developed countries in people aged over 60 years (Mitchell et al., 2018; Thomas et al., 2021). Damage to retinal pigment epithelial (RPE) cells caused by oxidative stress is thought to be a vital factor in the pathogenesis of AMD (Kaarniranta et al., 2020; Ruan et al., 2021; Toma et al., 2021). Recent research has also indicated that oxidative stress and RPE cell dysfunction are implicated in the pathogenesis of retinitis pigmentosa and other inherited retinal degenerative conditions (Gallenga et al., 2021; Pinilla et al., 2022). However, the exact mechanism by which oxidative stress causes damage to RPE cells is not comprehensively understood, and protecting RPE cells from oxidative stress-related damage remains a challenge.

Peroxiredoxin 6 (PRDX6) is a member of the non-selenium thiol peroxidase family, has both glutathione peroxidase and phospholipase A2 activities, and is widely distributed throughout many organs (e.g., the lungs, brain, liver, kidneys, and testes) and tissues (e.g., retina and optic nerve) (Fisher, 2011, 2017; Chidlow et al., 2016). There is growing evidence that PRDX6 acts as an antioxidant enzyme *in vivo* and can prevent oxidative stress (Manevich et al., 2002; Manevich and Fisher, 2005). In a previous study, we have found that PRDX6 levels were elevated in the retinas of rd12 mice, an animal model of Leber congenital amaurosis. This increase, a possible result of ongoing retinal degeneration, was normalized in the course of gene therapy, which led us to hypothesize that PRDX6 acts as an important antioxidant in the outer retina (Zheng et al., 2012). It is worth mentioning that the findings of Tulsawani et al. (2010) indicate that PRDX6 may play a similar role in the inner retina; they found that it provided protection against hypoxia-induced damage in retinal ganglion cells. Our hypothesis is also supported by Zha et al.’s (2015) more recent study in which it was shown that PRDX6 levels decreased after hydrogen peroxide (H_2_O_2_) treatment and that PRDX6 knockdown enhanced oxidative damage in RPE cells. In addition, another study showed that PRDX6, which is highly expressed in airway epithelial cells, suppressed lipopolysaccharide-induced Muc5ac via epidermal growth factor receptor (EGFR) and mitogen-activated protein kinases (MAPKs) (Yang et al., 2017). A recent study also showed PRDX6 protects irradiated cells from oxidative stress and shapes their senescence-associated cytokine landscape (Salovska et al., 2022). However, the presence of such a mechanism in RPE cells remains unclear, and the specific mechanism employed by PRDX6 to protect RPE cells from oxidative stress-induced damage has yet to be elucidated.

EGFR is located on the cell membrane, and its ligand is epidermal growth factor (EGF). When this tyrosine kinase receptor binds its ligand, it regulates cell growth, differentiation, and migration. When activated, EGFR can induce a series of intracellular transduction signaling events, such as activation of the following important cellular proteins: extracellular signal-regulated kinase (ERK), p38 mitogen-activated protein kinase (P38MAPK), and c-Jun N-terminal protein kinase (JNK) (Lemmon and Schlessinger, 2010). It was reported that PRDX6 expression in melanoma cells is maintained in a post-transcriptional manner by EGFR-dependent signaling, implying a link between PRDX6 and EGFR signaling (Schmitt et al., 2015). Additionally, a link between MAPKs and PRDX6 has been suggested by a report of MAPKs mediating phosphorylation of PRDX6 in alveolar cells (Wu et al., 2009). However, it remains unclear whether PRDX6 interferes with the EGFR/MAPK signaling pathway in RPE cells under oxidative stress.

In the present study, we investigated the effect of oxidative stress on the EGFR/MAPK signaling pathway in RPE cells and focused on the protective role that PRDX6 could play in mitigating the negative effects of oxidative stress. Our results show that damage to the RPE caused by H_2_O_2_-induced oxidative stress can be attenuated by PRDX6 overexpression via stimulation of the EGFR/ERK pathway. These findings suggest that PRDX6, with its antioxidant function, may protect RPE cells against oxidative stress-related damage.

## 2. Materials and methods

### 2.1. Cell culture and reagents

Cells from the ARPE-19 human RPE cell line were purchased from the American Type Culture Collection (ATCC, Manassas, VA, USA). The H_2_O_2_, 3-(4,5-dimethyl-2-thiazolyl)-2,5-diphenyl-2H- tetrazolium bromide (MTT), 2′,7′-dichlorodihydrofluorescein diacetate (DCFDA), and FluoroShield with Diamidinophenyl indole (DAPI) were purchased from Sigma-Aldrich (St. Louis, MO, USA). EGF was purchased from Peprotech Inc. (Rocky Hill, NJ, USA). Anti-PRDX6 primary antibody was purchased from Abcam Inc. (Cambridge, MA, USA). Anti-EGFR, anti-P38MAPK, anti-ERK, and anti-JNK primary antibodies were purchased from Cell Signaling Technology (Danvers, MA, USA).

### 2.2. Plasmid construction

The pLV-Flag PRDX6 plasmid was constructed from the full-size human PRDX6 cDNA sequence (PRDX6; GenBank accession no. NM_004905.2), which was provided as a gift by the Han Lab (http://hanlab.xmu.edu.cn/) from the School of Life Sciences at Xiamen University. The PRDX6 cDNA sequence was cut using restriction enzymes (*Sal*I and *Xma*I) and then assembled in a pLV-Flag vector.

### 2.3. Lentivirus-mediated PRDX6 overexpression

HEK293T cells (a human embryonic kidney cell line, ATCC CRL-3216) were transfected with a control plasmid, the pLV-Flag PRDX6 plasmid, and lentiviral packaging vectors. At 48 h post-transfection, the virus-containing supernatant was collected and used for further infection. Next, the ARPE-19 cells (2.0 × 10^5^ cells/mL) were cultured in culture media with the virus-containing supernatant and 10 μg/mL polybrene media for a further 48 h.

### 2.4. Cell viability assay

ARPE-19 cells were transferred to 96-well plates, treated with H_2_O_2_ for 6 h, and then incubated in a medium containing 50 μg/mL MTT for 4 h. Then, the medium was discarded, and the cells were fragmented using dimethyl sulfoxide. The absorbance value was measured at 550 nm using a microplate reader (POLARstar Omega, BMG Lab-tech, Germany).

### 2.5. Flow cytometric analysis of cell death

Briefly, after being treated with H_2_O_2_, ARPE-19 cells were gently trypsinized and washed twice with phosphate-buffered saline (PBS). Then, the cells were resuspended in a binding buffer and incubated with 5 μL annexin V-FITC and propidium iodide (PI) dyes (Keygen Inc., Nanjing, China) for 10 min. Finally, the cells were detected using a flow cytometer (Beckman Coulter Inc., Brea, CA, USA).

### 2.6. Reactive oxygen species (ROS) detection

Intracellular ROS production was detected as outlined in our previous study (Chen et al., 2016). Briefly, ARPE-19 cells (1.0 × 10^5^ cells/well) were treated with 100, 300, or 500 μM H_2_O_2_ for 6 h and incubated with 10 μM DCFDA for 20 min. DCFDA is converted into fluorescent 2′,7′-dichlorofluorescein by ROS (Eruslanov and Kusmartsev, 2010). After the cells were incubated with DCFDA, they were washed in PBS once and gently trypsinized. Finally, the cells were washed once again with PBS, suspended again in 1 mL PBS, and detected using the flow cytometer.

### 2.7. Western blot analysis

In brief, after ARPE-19 cells were rinsed twice with PBS and lysed with sodium dodecyl sulfate buffer, the proteins were denatured by incubating the samples at 100°C for 10 min. Equal proteins were loaded and separated on polyacrylamide gels, transferred to polyvinylidene difluoride (PVDF) membranes, and blocked with 5% milk for 2 h. Then, the PVDF membranes were rinsed thrice with PBST and incubated with primary antibodies (1:1,000) at 4°C overnight. Next, the PVDF membranes were rinsed thrice with PBST, incubated with secondary antibodies (1:2,000) at room temperature for 2 h, rinsed thrice with PBST, and stained with a chemiluminescence solution. Finally, the protein bands on the PVDF membranes were detected using the Bio-Rad ChemiDoc XRS system (Bio-Rad, Hercules, CA, USA).

### 2.8. Immunofluorescence

Briefly, after treatment with different reagents, cells on slides were fixed with 4% paraformaldehyde for 15 min, rinsed thrice with PBS, and then permeabilized by the addition of 0.1% Triton X-100 solution. They were then rinsed thrice in PBS and blocked with 5% bovine serum albumin solution for 1 h. Next, the cells were incubated with primary antibodies (1:100) at room temperature for 2 h, rinsed thrice in PBS, and incubated with fluorescent secondary antibodies (1:1,000) for 1 h. Finally, the cells were rinsed twice in PBS, shielded by FluoroShield with DAPI, and detected using a laser confocal microscope (Carl Zeiss, Jena, Germany).

### 2.9. Statistical analysis

All values are shown as mean ± SD or mean ± SEM. Statistical analysis was performed using GraphPad Prism 6 (GraphPad Software, LaJolla, CA, USA). Unpaired t-test was used to analyze differences between two groups. One-way ANOVA followed by Dunnett test was used to analyze differences among three or more groups. Significance is indicated as follows: *P < 0.05 and **P < 0.01.

## 3. Results

### 3.1. H_2_O_2_ induces damage in ARPE-19 cells

**FIGURE 1:**
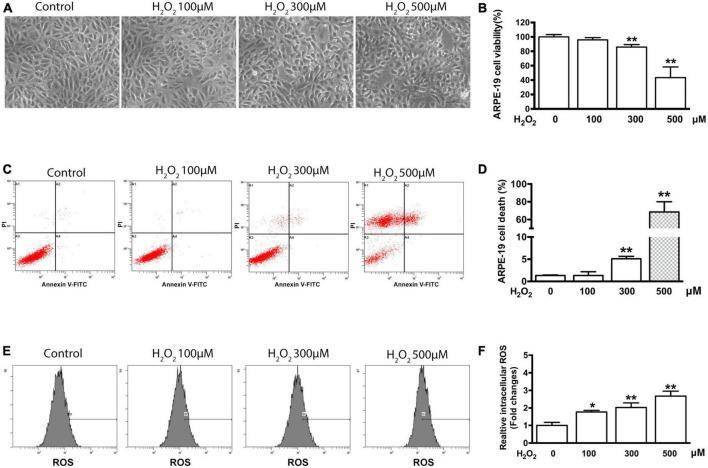
H_2_O_2_-induced oxidative damage in ARPE-19 cells. **(A)** Representative photomicrographs of ARPE-19 cells after application of 0, 100, 300, and 500 μM H_2_O_2_ for 6 h. Scale bar = 50 μm. **(B)** ARPE-19 cell viability was measured using an MTT assay, *n* = 12. **(C)** Flow cytometry results from the same experiment presented in panel **(A)**. **(D)** Quantification of cell death of sample shown in panel **(C)**, *n* = 3. **(E)** Flow cytometry was used to estimate ROS production in ARPE-19 cells using staining with DCFDA. **(F)** Bar graphs representing relative fluorescence densities of DCF, which was transformed from DCFDA by the ROS production shown in panel **(E)**. All data are means ± SD, *P < 0.05, **P < 0.01, compared to the control.

Cell shrinkage was observed by light microscopy in ARPE-19 cells treated with 300 and 500 μM H_2_O_2_ for 6 h; however, no shrinkage was observed in cells treated with 100 μM H_2_O_2_ (Figure 1A). To ascertain whether the observed cell shrinkage resulted in cell death and to quantify the cell death that occurred at each H_2_O_2_ concentration, the MTT assay and flow cytometry were used. The MTT assay results showed that, compared to control samples, samples treated with 100 μM H_2_O_2_ for 6 h did not exhibit a significant difference in cell viability. In contrast, treatment with either 300 or 500 μM H_2_O_2_ significantly reduced ARPE-19 cell viability (*P* < 0.01) under the same conditions. Furthermore, treatment with 500 μM H_2_O_2_ induced significantly more cell death than treatment with 300 μM H_2_O_2_ (56.6 vs. 14.1%, *P* < 0.01) (Figure 1B). The cytotoxic effect of the induced oxidative stress state on the cells was also determined using flow cytometry. The flow cytometry results confirmed the MTT assay results: after 6 h of treatment with 100 μM H_2_O_2_ no significant cell death was noted compared to the control condition (1.3% ± 0.9% vs. 1.3% ± 0.1%, *P* > 0.05), whereas treatment with 300 μM and 500 μM H_2_O_2_ caused significant cell death (5.1% ± 0.5% and 68.6% ± 11.6%, respectively, *P* < 0.01) (Figures 1C, D).

To elucidate the influence of some intracellular mechanisms that occur during oxidative stress on cell viability, intracellular ROS production was measured using flow cytometry and DCFDA dye. As shown in Figure 1E, a significant increase in ROS production was detected in the cells treated for 6 h with 100, 300, and 500 μM H_2_O_2_ compared with the control cells. Specifically, the relative level of ROS increased by 1.76 ± 0.1 (*P* < 0.05), 2.03 ± 0.26 (*P* < 0.01), and 2.68 ± 0.28 (*P* < 0.01) times, respectively (Figure 1F).

### 3.2. H_2_O_2_ affects EGFR and PRDX6 expression in ARPE-19 cells

**FIGURE 2:**
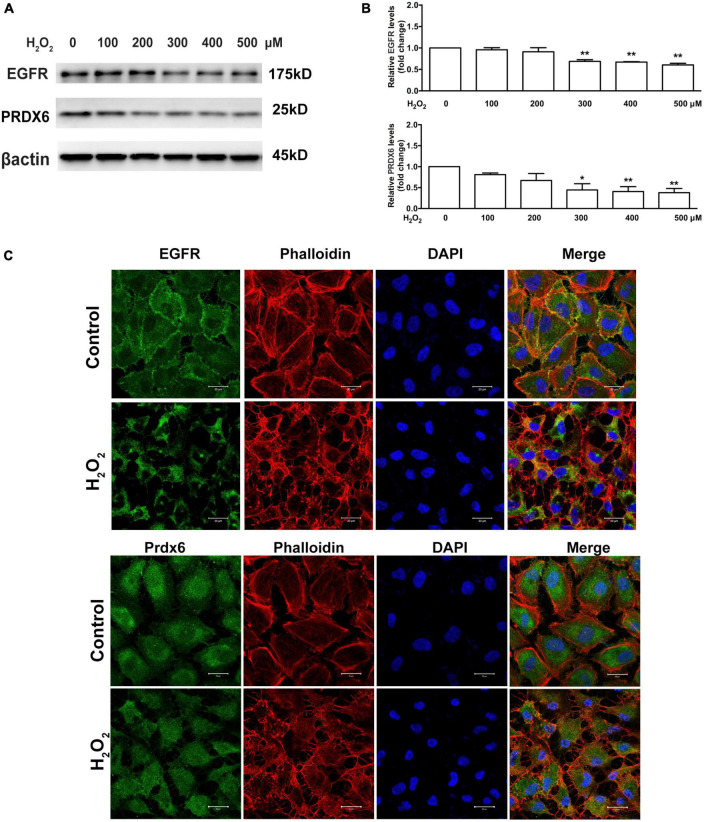
Oxidative stress affects EGFR and PRDX6 in ARPE-19 cells. **(A)** Western blot results of EGFR and PRDX6 after ARPE-19 cells were treated with different concentrations of H_2_O_2_ for 6 h. **(B)** Quantitative analysis of western blot results from three independent experiments. *P < 0.05, **P < 0.01, compared with the control. **(C)** Immunofluorescence staining of ARPE-19 cells after treatment with 300 μM H_2_O_2_ for 6 h. EGFR (green), PRDX6 (green), phalloidin (red), and DAPI (blue). Scale bar = 20 μm.

The levels of EGFR and PRDX6 were observed to be lower in cells treated with 100 and 200 μM H_2_O_2_; however, Western blot quantification showed that the differences were not statistically significant. In contrast, cells treated with 300, 400, and 500 μM H_2_O_2_ showed significant downregulation of EGFR and PRDX6 (25% or more for EGFR and 50% or more for PRDX6, *P* < 0.05) (Figures 2A, B). Additionally, immunofluorescence staining showed that treatment with 300 μM H_2_O_2_ for 6 h induced cell membrane-bound EGFR to translocate into the cytoplasm and clearly decreased the expression of both EGFR and PRDX6. Staining with the fluorescent phalloidin dye showed cell body shrinkage and cytoskeleton destruction, thus providing indirect confirmation of loss of cell viability. This was further supported by the shrinkage of all cell nuclei observed with DAPI-staining (Figure 2C).

### 3.3. H_2_O_2_ affects the EGFR/MAPK signaling pathway in ARPE-19 cells

**FIGURE 3:**
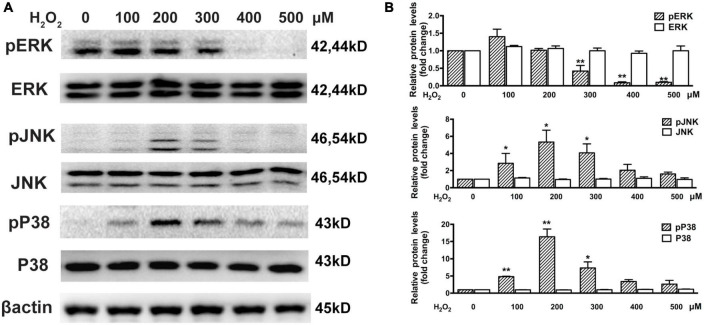
Oxidative stress affects MAPKs in ARPE-19 cells. **(A)** Western blot results show ARPE-19 cells treated with H_2_O_2_ for 6 h. Total and phosphorylated P38MAPK, ERK, and JNK were detected. **(B)** Quantitative analysis of western blot results from three independent experiments. *P < 0.05, **P < 0.01, compared to control.

**FIGURE 4:**
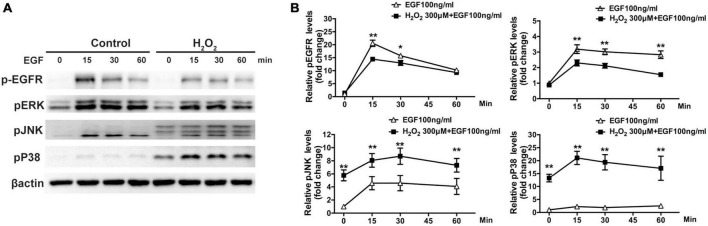
Oxidative stress affects EGF-induced EGFR/MAPK signaling in ARPE-19 cells. **(A)** ARPE-19 cells were treated with or without 300 μM H_2_O_2_ for 3 h and then treated with 100 ng/mL EGF for 15, 30, or 60 min. Phosphorylated EGFR, P38MAPK, ERK, JNK, and β-actin were detected by western blot assay. **(B)** Quantitative analysis and comparison of western blot assay results, *P < 0.05, **P < 0.01.

The Western blot assay results show that there was significant downregulation of phosphorylated ERK in cells treated with 300–500 μM H_2_O_2_ and significant upregulation of phosphorylated P38MAPK (approximately 5–15 times) and JNK (approximately 2.5–5 times) in cells treated with 100–300 μM H_2_O_2_ (Figure 3). Furthermore, we estimated the effects of H_2_O_2_ on EGF-induced EGFR/MAPK signaling using a Western blot assay. For this, ARPE-19 cells were pretreated with 300 μM H_2_O_2_ for 3 h, and then the cells were treated with 100 ng/mL EGF for 15, 30, or 60 min. The results show that EGF induced significant EGFR, ERK, and JNK phosphorylation, as well as a small amount of P38MAPK phosphorylation. In contrast, pretreatment with 300 μM H_2_O_2_ for 3 h did not induce significant EGFR and ERK phosphorylation; however, it caused significant P38MAPK and JNK phosphorylation. Interestingly, the EGF-induced phosphorylation of EGFR and ERK was significantly inhibited by pretreatment with H_2_O_2_ (Figures 4A, B).

### 3.4. PRDX6 overexpression attenuates H_2_O_2_-induced damage in ARPE-19 cells

**FIGURE 5:**
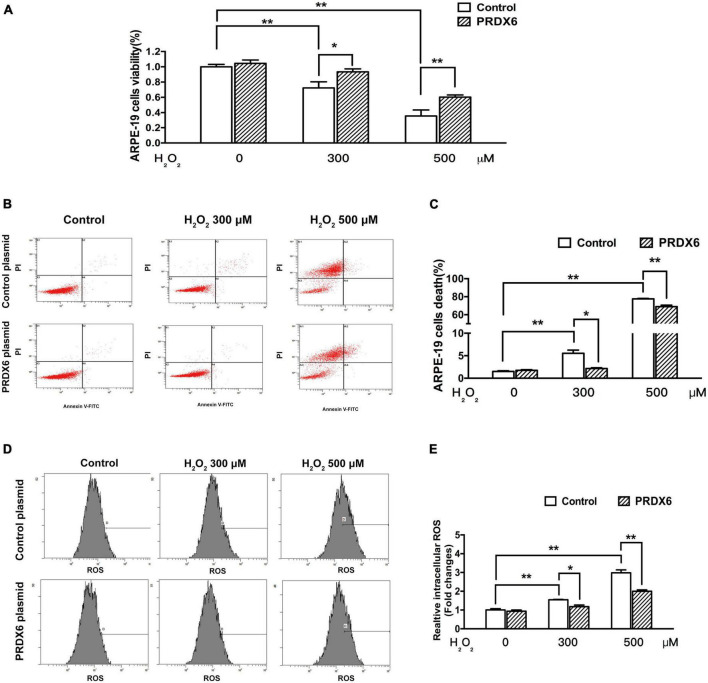
PRDX6 overexpression attenuates H_2_O_2_-induced oxidative damage in ARPE-19 cells. ARPE-19 cells were infected with lentivirus containing a control plasmid or the pLV-Flag PRDX6 plasmid for 48 h and were then treated with H_2_O_2_. **(A)** Cell viability was measured using an MTT assay. **(B)** Cell death analysis by flow cytometry. **(C)** Quantification of ARPE-19 cell death, *n* = 3. **(D)** Flow cytometric analysis of ROS levels, with DCFDA staining. **(E)** Histograms representing the relative ROS levels, which were fluorescence densities of DCF transformed from DCFDA, *n* = 3, *P < 0.05, **P < 0.01.

The ability of PRDX6 to protect ARPE-19 cells from H_2_O_2_-induced damage was examined using an MTT assay and flow cytometry. The MTT assay results show that PRDX6 overexpression effectively reduced the amount of cell death induced by treatment with 300 and 500 μM H_2_O_2_ for 6 h, with the level of cell death reduced by about 21.1% (*P* < 0.05) and 24.6% (*P* < 0.01), respectively (Figure 5A). Similarly, the flow cytometry results show that PRDX6 overexpression decreased the amount of cell death induced by treatment with 300 and 500 μM H_2_O_2_ for 6 h by about 3.4% (*P* < 0.05) and 8.6% (*P* < 0.01), respectively (Figures 5B, C). Furthermore, the flow cytometric analysis of cells stained with DCFDA showed that PRDX6 overexpression significantly attenuated the high ROS levels induced by treatment with 300 and 500 μM H_2_O_2_ for 6 h (Figures 5D, E).

### 3.5. PRDX6 overexpression affects EGFR and ERK expression in ARPE-19 cells

**FIGURE 6:**
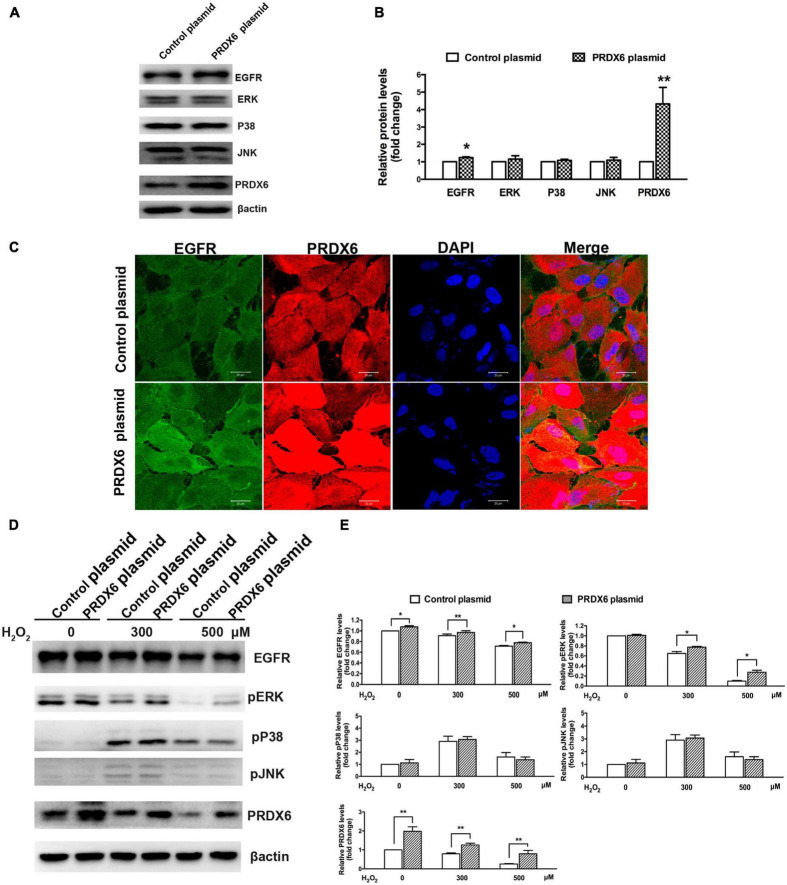
The effect of PRDX6 overexpression on EGFR and MAPKs in ARPE-19 cells. **(A)** Cells were infected with lentivirus containing the PRDX6 plasmid. Total EGFR, P38MAPK, ERK, JNK, and β-actin were detected by western blot. **(B)** Quantitative analysis of western blot results, *P < 0.05, **P < 0.01, compared to control. **(C)** Immunofluorescence staining of EGFR, PRDX6, and DAPI after ARPE-19 cells were infected with lentivirus containing a control plasmid or the pLV-Flag PRDX6 plasmid. Scale bar = 20 μm. **(D)** Cells were infected with lentivirus containing a control plasmid or the pLV-Flag PRDX6 plasmid for 48 h and were treated with 300 and 500 μM H_2_O_2_ for 6 h. EGFR, P38MAPK, ERK, JNK, and β-actin were detected by western blot. **(E)** Quantitative analysis of western blot results is shown in panel **(D)**. All data were from three independent experiments, *P < 0.05, **P < 0.01.

**FIGURE 7:**
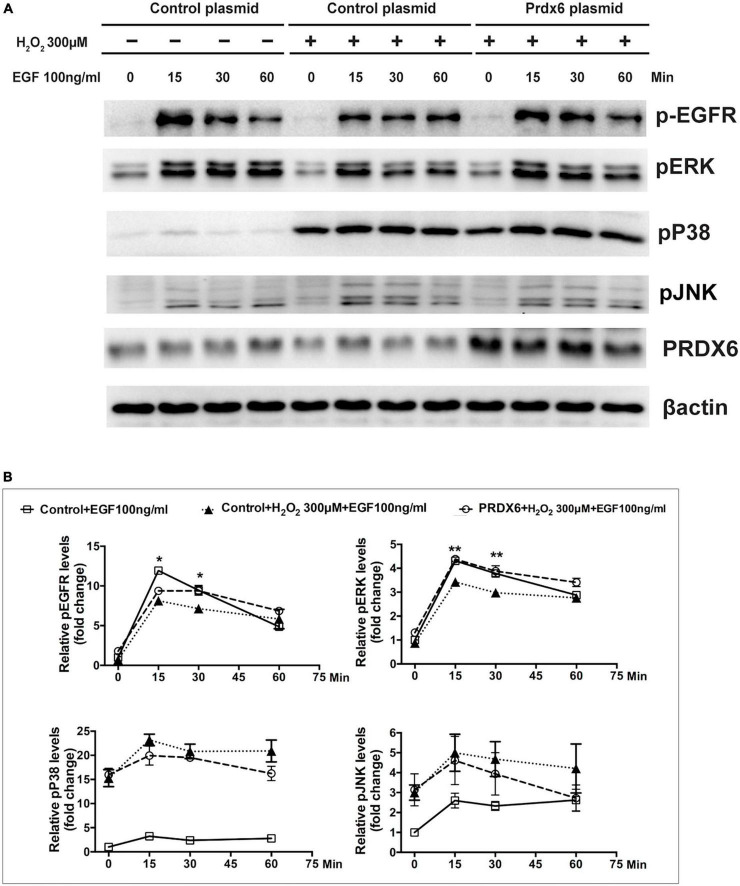
PRDX6 overexpression attenuates inhibition of EGF-induced EGFR/ERK signaling caused by H_2_O_2_. ARPE-19 cells were infected with lentivirus containing a plasmid for 48 h, pretreated with 300 μM H_2_O_2_ for 3 h, and then induced by application of 100 ng/mL EGF for 15, 30, or 60 min. **(A)** Phosphorylated EGFR, ERK, P38MAPK, JNK, and β-actin were detected by western blot. **(B)** Quantitative data of western blot results shown in panel **(A)**. Comparisons are among the control group, H_2_O_2_ treatment group, and PRDX6 overexpression combined with H_2_O_2_ treatment group at same time, *P < 0.05, **P < 0.01.

A Western blot assay was also conducted to determine the effect of overexpression of PRDX6 on EGFR and ERK expression in ARPE-19 cells. The findings show that overexpression of PRDX6 increased the expression of EGFR by about 24% (*P* < 0.01); however, it did not cause significant changes in the expression of ERK, P38MAPK, and JNK (Figures 6A, B). The results of an immunofluorescence image analysis confirmed that PRDX6 overexpression caused an increase in EGFR expression (Figure 6C). Furthermore, we investigated the effects of PRDX6 overexpression on EGFR and MAPKs in ARPE-19 cells exposed to H_2_O_2_-induced oxidative stress. The results show that PRDX6 overexpression partially attenuated the H_2_O_2_-induced decrease in EGFR and phosphorylated ERK. However, it did not affect the H_2_O_2_-induced increase in phosphorylated P38MAPK and phosphorylated JNK (Figures 6D, E). Moreover, the Western blot results show that EGF induced significant EGFR, ERK, and JNK phosphorylation, and slight P38MAPK phosphorylation. In contrast, pretreatment with 300 μM H_2_O_2_ for 3 h led to significant phosphorylation of P38MAPK and JNK, whereas it inhibited EGFR and ERK phosphorylation caused by EGF. While PRDX6 overexpression attenuated H_2_O_2_-caused inhibition of EGFR and ERK phosphorylation induced by EGF, it did not affect H_2_O_2_-caused P38MAPK and JNK phosphorylation in ARPE-19 cells (Figures 7A, B).

## 4. Discussion

Extensive research by ophthalmologists and scientists has been focused on investigating the damage caused by oxidative stress in RPE cells (Cai et al., 2000; Lu et al., 2006; Koinzer et al., 2015), particularly in the context of AMD (Totsuka et al., 2019; Subramaniam et al., 2020; Terao et al., 2022). Recent studies have indicated that oxidative stress-related damage in RPE cells could lead to the degeneration of photoreceptor cells and plays a vital role in the pathogenesis of hereditary retinal diseases, including retinitis pigmentosa and other inherited photoreceptor degenerative conditions (Gallenga et al., 2021; Pinilla et al., 2022; Vingolo et al., 2022). However, the detailed molecular mechanism that mediates oxidative stress-related damage in RPE cells is not fully understood.

Oxidative stress induced by H_2_O_2_ often results in classical oxidative damage in RPE cells (Ho et al., 2006; Kaczara et al., 2010; Cui et al., 2020), and some studies have shown that antioxidants can efficiently maintain the viability of RPE cells in a state of oxidative stress (Cheng et al., 2014; Subramanian et al., 2016; Demirci Kucuk et al., 2022). In the present research, we mostly used three concentrations of H_2_O_2_ (100, 300, and 500 μM) to induce oxidative stress, and we used the fluorescent dye DCFDA to detect ROS in ARPE-19 cells. Our results show that H_2_O_2_ at these concentrations induced a detectable increase in ROS levels at 6 h post-treatment (Figures 1E, F). Although the slight oxidative stress caused by 100 μM H_2_O_2_ did not induce significant inhibition of cell viability or increase cell death, moderate and severe oxidative stress caused by 300 and 500 μM H_2_O_2_, respectively, caused significant cell viability inhibition and cell death. Under physiological conditions (*in vivo*), ROS are formed either during metabolic processes or when organisms are exposed to various stress factors. The effects of oxidative stress mainly depend on the intensity of the ROS-induced oxidative damage and the cellular response to this oxidative damage (Halliwell, 2013; Fanjul-Moles and López-Riquelme, 2016). Our results suggest that ROS accumulated in the cells treated with 300 and 500 μM at levels that exceeded the limit of the normally tolerated concentration of intracellular ROS (likely below the equivalent of ROS generated by 100 μM H_2_O_2_) and that this might be an important activator of REP cell dysfunction.

As a well-established model of oxidative stress in RPE cells, recent reports have shown that H_2_O_2_ may be used in working concentrations from 100 to 1,800 μM. Ayala-Peña et al. found that treatment with 500 and 750 μM H_2_O_2_ for 24 h induced significant inhibition of RPE cell viability (Ayala-Peña et al., 2016). Others have reported that RPE cells exposed to 500 μM H_2_O_2_ for 24 h exhibited a significant decrease in cell viability (Du et al., 2017), and treatment with 100–1,800 μM H_2_O_2_ has been shown to inhibit RPE cell viability in a concentration-dependent manner after 24 h (Zhu et al., 2017). In this study, there was no significant cell death found in the treatment of 100 μM H_2_O_2_ for 6 hours by the flow cytometry analysis. However, with the increasing of H_2_O_2_ treatment to 300 μM and 500 μM, minimal cell death and significant cell death were found respectively. Taken together, these results from previous studies and our current study suggest that moderate concentrations of H_2_O_2_ (100–300 μM) may better reflect a physiologically relevant cellular environment, while higher concentrations of H_2_O_2_ may result in pronounced cytotoxicity and therefore be less representative of physiological and pathophysiological processes that occur *in vivo*. Regarding the mechanism underlying RPE cell death induced by H_2_O_2_, previous studies have indicated that apoptosis is the primary mechanism involved (Jin et al., 2001; Kim et al., 2003). In addition, others have found that necrosis is also an important form of RPE cell death in oxidative stress (Hanus et al., 2013; Zhu et al., 2016). In the present study, the flow cytometric analysis showed that there was a significant increase in the percentage of PI-stained cells in the samples treated with 500 μM H_2_O_2_ for 6 h; however, there was no significant increase in the percentage of annexin V-stained cells in the same samples (Figure 1E). These results further support the idea that necrosis might be a significant contributor to RPE cell dysfunction in response to severe oxidative stress.

In the present study, treatment with H_2_O_2_ resulted in a significant decrease in EGFR, PRDX6, and phosphorylated ERK levels, whereas it induced JNK and P38MAPK phosphorylation. The presence of EGFR in the cytomembrane also plays a vital role in ocular development and photoreceptor differentiation (Zhang and Du, 2015; Malartre, 2016). The EGFR signaling pathway can initiate cell growth, division, migration, and proliferation (Yan et al., 2007; Zhang et al., 2013, 2022). MAPKs, particularly JNK, P38MAPK, and ERK, are crucial downstream proteins of EGFR signaling, and, thereby play a critical role in a series of physiological cellular activities, such as RPE cell proliferation, differentiation, and migration (Samuel et al., 2008; Maugeri et al., 2019; Li et al., 2021). In several studies, phosphorylated ERK in RPE cells has been shown to be increased by treatment with H_2_O_2_ (Qin et al., 2006; Wankun et al., 2011). On the other hand, some studies showed the phosphorylation level of ERK1/2 was significantly inhibited by H_2_O_2_ (He et al., 2014; Chen et al., 2021). Although these results seem paradoxical, we think that these results are not mutually exclusive. We found that after RPE cells were treated with 300 μM H_2_O_2_ for different periods of time, phosphorylated ERK was elevated for a short period after treatment, while it was significantly decreased for a long period after treatment (Supplementary Figures 1A, B). We speculate one possible reason for this phenomenon is that treatment with a lower concentration of H_2_O_2_ can activate phosphorylated ERK after a short period, while treatment with a higher concentration of H_2_O_2_ can inhibit phosphorylated ERK for a relatively long period.

PRDX6 is a cytoprotective, dual-functional enzyme with both glutathione peroxidase and phospholipase A2 activities (Arevalo and Vázquez-Medina, 2018; Wahlig et al., 2018). An increasing number of studies indicate that PRDX6 can protect cells from oxidative stress-related injury by reducing phospholipid hydroperoxides, controlling ROS levels, and other mechanisms (Kubo et al., 2008; Wang et al., 2008; Asuni et al., 2015). Zha et al. reported that PRDX6 might protect ARPE-19 cells from oxidative damage and apoptosis induced by H_2_O_2_ via the PI3K/AKT signaling pathway (Zha et al., 2015). Another study showed that PRDX6 protected crystalline lens cells against oxidative stress induced by UV exposure by reducing the levels of ROS (Shibata et al., 2016). Our results show that oxidative stress induced by H_2_O_2_ led to a significant decrease in PRDX6 levels. We also found that overexpression of PRDX6 significantly attenuated ARPE-19 cell viability inhibition, cell death, and ROS production induced by H_2_O_2_. Overall, our results, combined with results from previous studies, suggest that PRDX6 might be an important target of oxidative stress and a key regulator of redox balance in RPE cells. These findings could also provide a foundation for the development of an antioxidant-based gene therapy for some retinal degenerative diseases in which oxidative damage in RPE cells contributes to pathogenesis.

Some studies have showed that PRDX6 was related to the EGFR signaling pathway. Schmitt et al. (2015) found that PRDX6 is elevated in melanoma cells in an EGFR-dependent manner and is an important driver of cell proliferation. Others have also reported that PRDX6 may regulate LPS-induced airway inflammation by suppressing the Muc5ac overproduction induced by LPS via the H_2_O_2_-EGFR-MAPK pathway (Yang et al., 2017). Our findings revealed overexpression of PRDX6 resulted in increased EGFR levels, attenuated reductions of EGFR and phosphorylated ERK caused by H_2_O_2_. Moreover, the oxidative stress-related inhibition of EGFR and ERK phosphorylation caused by EGF was also alleviated by PRDX6 overexpression. Overall, these results suggest that EGFR/MAPK signaling is involved in oxidative damage in RPE cells and that PRDX6 might reduce oxidative damage by inducing the EGFR/ERK signaling pathway. There are still some limitations to consider in this study. Firstly, the *in vitro* stimulation of H_2_O_2_-induced oxidative stress may not fully replicate the complex cellular environment found *in vivo*. Treatment with a lower concentration of H_2_O_2_ over an extended period of time would provide a more accurate reflection of the *in vivo* conditions. In addition, further *in vivo* studies should be conducted to determine whether PRDX6 can protect RPE cells and photoreceptor cells against damage induced by endogenous and exogenous oxidative stress.

In conclusion, our findings demonstrated that the H_2_O_2_ exposure could lead to significant oxidative stress-related damage in RPE cells. Moreover, our study reveals that overexpression of PRDX6 overexpression protect ARPE-19 cells against oxidative stress through activation of the EGFR/ERK signaling pathway. These findings shed new light on the protective role of PRDX6 in RPE cells and suggest its potential role in the development of protectants and therapeutics for retinal degenerative diseases such as AMD.

## Data availability statement

The datasets presented in this study can be found in online repositories. The names of the repository/repositories and accession number(s) can be found below: https://www.ncbi.nlm.nih.gov/genbank/, NCBI Reference Sequence: NM_004905.2.

## Author contributions

WL, AH, and XC conceived and designed the experiments. XC, MS, and YZ performed the experiments. XC and WL analyzed the data. WL and AH contributed reagents, materials, and analysis tools. XC and RT wrote the manuscript. All authors contributed to the manuscript and approved the submitted version.

## Supplementary material

Supplementary Figure 1 Treatment with 300 μM H_2_O_2_ for different times affects pERK in ARPE-19 cells. **(A)** Western blot results of pERK and ERK after ARPE-19 cells were treated with 300 μM H_2_O_2_ for 0, 0.25, 0.5, 1, 3 or 6 h. **(B)** Quantitative analysis of western blot results from three independent experiments. **P* < 0.05, ***P* < 0.01, compared to control.

## References

[B1] Arevalo J. A. Vázquez-Medina J. P. (2018). The role of peroxiredoxin 6 in cell signaling. *Antioxidants* 7:172. 10.3390/antiox7120172 30477202 PMC6316032

[B2] Asuni A. A. Guridi M. Sanchez S. Sadowski M. J. (2015). Antioxidant peroxiredoxin 6 protein rescues toxicity due to oxidative stress and cellular hypoxia in vitro, and attenuates prion-related pathology in vivo. *Neurochem. Int.* 90 152–165. 10.1016/j.neuint.2015.08.006 26265052 PMC4641785

[B3] Ayala-Peña V. B. Pilotti F. Volonté Y. Rotstein N. P. Politi L. E. German O. L. (2016). Protective effects of retinoid x receptors on retina pigment epithelium cells. *Biochim. Biophys. Acta* 1863 1134–1145. 10.1016/j.bbamcr.2016.02.010 26883505

[B4] Cai J. Nelson K. C. Wu M. Sternberg P. Jr. Jones D. P. (2000). Oxidative damage and protection of the RPE. *Prog. Retin. Eye. Res.* 19 205–221. 10.1016/s1350-9462(99)00009-9 10674708

[B5] Chen J. Wang D. Zong Y. Yang X. (2021). DHA protects hepatocytes from oxidative injury through GPR120/ERK-mediated mitophagy. *Int. J. Mol. Sci.* 22:5675. 10.3390/ijms22115675 34073582 PMC8198367

[B6] Chen X. Tzekov R. Su M. Hong H. Min W. Han A. (2016). Auranofin inhibits retinal pigment epithelium cell survival through reactive oxygen species-dependent epidermal growth factor receptor/mitogen-activated protein kinase signaling pathway. *PLoS One* 11:e0166386. 10.1371/journal.pone.0166386 27846303 PMC5112952

[B7] Cheng L. B. Chen C. M. Zhong H. Zhu L. J. (2014). Squamosamide derivative FLZ protects retinal pigment epithelium cells from oxidative stress through activation of epidermal growth factor receptor (EGFR)-AKT signaling. *Int. J. Mol. Sci.* 15 18762–18775. 10.3390/ijms151018762 25329617 PMC4227245

[B8] Chidlow G. Wood J. P. Knoops B. Casson R. J. (2016). Expression and distribution of peroxiredoxins in the retina and optic nerve. *Brain Struct. Funct.* 221 3903–3925. 10.1007/s00429-015-1135-3 26501408 PMC5065902

[B9] Cui R. Tian L. Lu D. Li H. Cui J. (2020). Exendin-4 protects human retinal pigment epithelial cells from H_2_O_2_-induced oxidative damage via activation of NRF2 signaling. *Ophthalmic Res.* 63 404–412. 10.1159/000504891 31865348

[B10] Demirci Kucuk K. Tokuc E. O. Aciksari A. Duruksu G. Yazir Y. Karabas V. L. (2022). The effects of crocetin on oxidative stress induced ARPE-19 cells by H_2_O_2_. *Exp. Eye Res.* 226:109305. 10.1016/j.exer.2022.109305 36372214

[B11] Du L. Chen J. Xing Y. Q. (2017). Eupatilin prevents H_2_O_2_-induced oxidative stress and apoptosis in human retinal pigment epithelial cells. *Biomed. Pharmacother.* 85 136–140. 10.1016/j.biopha.2016.11.108 27930977

[B12] Eruslanov E. Kusmartsev S. (2010). Identification of ROS using oxidized DCFDA and flow-cytometry. *Methods Mol. Biol.* 594 57–72. 10.1007/978-1-60761-411-1_4 20072909

[B13] Fanjul-Moles M. L. López-Riquelme G. O. (2016). Relationship between oxidative stress, circadian rhythms, and AMD. *Oxid. Med. Cell Longev.* 2016:7420637. 10.1155/2016/7420637 26885250 PMC4738726

[B14] Fisher A. B. (2011). Peroxiredoxin 6: a bifunctional enzyme with glutathione peroxidase and phospholipase A2 activities. *Antioxid. Redox Signal.* 15 831–844. 10.1089/ars.2010.3412 20919932 PMC3125547

[B15] Fisher A. B. (2017). Peroxiredoxin 6 in the repair of peroxidized cell membranes and cell signaling. *Arch. Biochem. Biophys.* 617 68–83. 10.1016/j.abb.2016.12.003 27932289 PMC5810417

[B16] Gallenga C. E. Lonardi M. Pacetti S. Violanti S. S. Tassinari P. Di Virgilio F. (2021). Molecular mechanisms related to oxidative stress in retinitis pigmentosa. *Antioxidants* 10:848. 10.3390/antiox10060848 34073310 PMC8229325

[B17] Halliwell B. (2013). The antioxidant paradox: less paradoxical now? *Br. J. Clin. Pharmacol.* 75 637–644. 10.1111/j.1365-2125.2012.04272.x 22420826 PMC3575931

[B18] Hanus J. Zhang H. Wang Z. Liu Q. Zhou Q. Wang S. (2013). Induction of necrotic cell death by oxidative stress in retinal pigment epithelial cells. *Cell Death Dis.* 4:e965. 10.1038/cddis.2013.478 24336085 PMC3877549

[B19] He Y. Leung K. Ren Y. Pei J. Ge J. Tombran-Tink J. (2014). PEDF improves mitochondrial function in RPE cells during oxidative stress. *Invest. Ophthalmol. Vis. Sci.* 55 6742–6755. 10.1167/iovs.14-14696 25212780

[B20] Ho T. C. Yang Y. C. Cheng H. C. Wu A. C. Chen S. L. Chen H. K. (2006). Activation of mitogen-activated protein kinases is essential for hydrogen peroxide -induced apoptosis in retinal pigment epithelial cells. *Apoptosis* 11 1899–1908. 10.1007/s10495-006-9403-6 16927023

[B21] Jin G. F. Hurst J. S. Godley B. F. (2001). Hydrogen peroxide stimulates apoptosis in cultured human retinal pigment epithelial cells. *Curr. Eye Res.* 22 165–173. 10.1076/ceyr.22.3.165.5517 11462152

[B22] Kaarniranta K. Uusitalo H. Blasiak J. Felszeghy S. Kannan R. Kauppinen A. (2020). Mechanisms of mitochondrial dysfunction and their impact on age-related macular degeneration. *Prog. Retin. Eye Res.* 79:100858. 10.1016/j.preteyeres.2020.100858 32298788 PMC7650008

[B23] Kaczara P. Sarna T. Burke J. M. (2010). Dynamics of H_2_O_2_ availability to ARPE-19 cultures in models of oxidative stress. *Free Radic. Biol. Med.* 48 1064–1070. 20100568 10.1016/j.freeradbiomed.2010.01.022 PMC2839027

[B24] Kim M. H. Chung J. Yang J. W. Chung S. M. Kwag N. H. Yoo J. S. (2003). Hydrogen peroxide-induced cell death in a human retinal pigment epithelial cell line, ARPE-19. *Korean J. Ophthalmol.* 17 19–28. 10.3341/kjo.2003.17.1.19 12882504

[B25] Koinzer S. Reinecke K. Herdegen T. Roider J. Klettner A. (2015). Oxidative stress induces biphasic ERK1/2 activation in the RPE with distinct effects on cell survival at early and late activation. *Curr. Eye Res.* 40 853–857. 10.3109/02713683.2014.961613 25251900

[B26] Kubo E. Fatma N. Akagi Y. Beier D. R. Singh S. P. Singh D. P. (2008). TAT-mediated PRDX6 protein transduction protects against eye lens epithelial cell death and delays lens opacity. *Am. J. Physiol. Cell Physiol.* 294 C842–C855. 10.1152/ajpcell.00540.2007 18184874

[B27] Lemmon M. A. Schlessinger J. (2010). Cell signaling by receptor tyrosine kinases. *Cell* 141 1117–1134. 10.1016/j.cell.2010.06.011 20602996 PMC2914105

[B28] Li T. Zhang H. B. Meng J. M. Yuan B. Lin W. J. Feng Y. (2021). YM155 inhibits retinal pigment epithelium cell survival through EGFR/MAPK signaling pathway. *Int. J. Ophthalmol.* 14 489–496. 10.18240/ijo.2021.04.02 33875937 PMC8025172

[B29] Lu L. Hackett S. F. Mincey A. Lai H. Campochiaro P. A. (2006). Effects of different types of oxidative stress in RPE cells. *J. Cell Physiol.* 206 119–125. 10.1002/jcp.20439 15965958

[B30] Malartre M. (2016). Regulatory mechanisms of EGFR signalling during Drosophila eye development. *Cell Mol. Life Sci.* 73 1825–1843. 10.1007/s00018-016-2153-x 26935860 PMC11108404

[B31] Manevich Y. Fisher A. B. (2005). Peroxiredoxin 6, a 1-Cys peroxiredoxin, functions in antioxidant defense and lung phospholipid metabolism. *Free Radic Biol. Med.* 38 1422–1432. 10.1016/j.freeradbiomed.2005.02.011 15890616

[B32] Manevich Y. Sweitzer T. Pak J. H. Feinstein S. I. Muzykantov V. Fisher A. B. (2002). 1-Cys peroxiredoxin overexpression protects cells against phospholipid peroxidation-mediated membrane damage. *Proc. Natl. Acad. Sci.U.S.A.* 99 11599–11604. 10.1073/pnas.182384499 12193653 PMC129315

[B33] Maugeri G. D’Amico A. G. Bucolo C. D’Agata V. (2019). Protective effect of PACAP-38 on retinal pigmented epithelium in an in vitro and in vivo model of diabetic retinopathy through EGFR-dependent mechanism. *Peptides* 119:170108. 10.1016/j.peptides.2019.170108 31247223

[B34] Mitchell P. Liew G. Gopinath B. Wong T. Y. (2018). Age-related macular degeneration. *Lancet* 392 1147–1159. 10.1016/S0140-6736(18)31550-2 30303083

[B35] Pinilla I. Maneu V. Campello L. Fernández-Sánchez L. Martínez-Gil N. Kutsyr O. (2022). Inherited retinal dystrophies: role of oxidative stress and inflammation in their physiopathology and therapeutic implications. *Antioxidants* 11:1086. 10.3390/antiox11061086 35739983 PMC9219848

[B36] Qin S. McLaughlin A. De Vries G. (2006). Protection of RPE cells from oxidative injury by 15-deoxy-delta12,14-prostaglandin J2 by augmenting GSH and activating MAPK. *Invest. Ophthalmol. Vis. Sci.* 47 5098–5105. 10.1167/iovs.06-0318 17065531

[B37] Ruan Y. Jiang S. Gericke A. (2021). Age-related macular degeneration: role of oxidative stress and blood vessels. *Int. J. Mol. Sci.* 22:1296. 10.3390/ijms22031296 33525498 PMC7866075

[B38] Salovska B. Kondelova A. Pimkova K. Liblova Z. Pribyl M. Fabrik I. (2022). Peroxiredoxin 6 protects irradiated cells from oxidative stress and shapes their senescence-associated cytokine landscape. *Redox Biol.* 49:102212. 10.1016/j.redox.2021.102212 34923300 PMC8688892

[B39] Samuel W. Kutty R. K. Sekhar S. Vijayasarathy C. Wiggert B. Redmond T. M. (2008). Mitogen-activated protein kinase pathway mediates N-(4-hydroxyphenyl)retinamide-induced neuronal differentiation in the ARPE-19 human retinal pigment epithelial cell line. *J. Neurochem.* 106 591–602. 10.1111/j.1471-4159.2008.05409.x 18410500 PMC2694741

[B40] Schmitt A. Schmitz W. Hufnagel A. Schartl M. Meierjohann S. (2015). Peroxiredoxin 6 triggers melanoma cell growth by increasing arachidonic acid-dependent lipid signalling. *Biochem. J.* 471 267–279. 10.1042/BJ20141204 26285655

[B41] Shibata S. Shibata N. Shibata T. Sasaki H. Singh D. P. Kubo E. (2016). The role of Prdx6 in the protection of cells of the crystalline lens from oxidative stress induced by UV exposure. *Jpn. J. Ophthalmol.* 60 408–418. 10.1007/s10384-016-0461-1 27379999 PMC5544922

[B42] Subramaniam M. D. Iyer M. Nair A. P. Venkatesan D. Mathavan S. Eruppakotte N. (2020). Oxidative stress and mitochondrial transfer: a new dimension towards ocular diseases. *Genes Dis.* 9 610–637. 10.1016/j.gendis.2020.11.020 35782976 PMC9243399

[B43] Subramanian P. Mendez E. F. Becerra S. P. (2016). A novel inhibitor of 5-lipoxygenase (5-LOX) prevents oxidative stress-induced cell death of retinal pigment epithelium (RPE) cells. *Invest. Ophthalmol. Vis. Sci.* 57 4581–4588. 10.1167/iovs.15-19039 27635633 PMC5033602

[B44] Terao R. Ahmed T. Suzumura A. Terasaki H. (2022). Oxidative stress-Induced cellular senescence in aging retina and age-Related macular degeneration. *Antioxidants* 11:2189. 10.3390/antiox11112189 36358561 PMC9686487

[B45] Thomas C. J. Mirza R. G. Gill M. K. (2021). Age-related macular degeneration. *Med. Clin. North. Am.* 105 473–491. 10.1016/j.mcna.2021.01.003 33926642

[B46] Toma C. De Cillà S. Palumbo A. Garhwal D. P. Grossini E. (2021). Oxidative and nitrosative stress in age-related macular degeneration: a review of their role in different stages of disease. *Antioxidants* 10:653. 10.3390/antiox10050653 33922463 PMC8145578

[B47] Totsuka K. Ueta T. Uchida T. Roggia M. F. Nakagawa S. Vavvas D. G. (2019). Oxidative stress induces ferroptotic cell death in retinal pigment epithelial cells. *Exp. Eye Res.* 181 316–324. 10.1016/j.exer.2018.08.019 30171859 PMC7418497

[B48] Tulsawani R. Kelly L. S. Fatma N. Chhunchha B. Kubo E. Kumar A. (2010). Neuroprotective effect of peroxiredoxin 6 against hypoxia-induced retinal ganglion cell damage. *BMC Neurosci.* 11:125. 10.1186/1471-2202-11-125 20923568 PMC2964733

[B49] Vingolo E. M. Casillo L. Contento L. Toja F. Florido A. (2022). Retinitis pigmentosa (RP): the role of oxidative stress in the degenerative process progression. *Biomedicines* 10:582. 10.3390/biomedicines10030582 35327384 PMC8945005

[B50] Wahlig S. Lovatt M. Mehta J. S. (2018). Functional role of peroxiredoxin 6 in the eye. *Free. Radic. Biol. Med.* 126 210–220. 30120980 10.1016/j.freeradbiomed.2018.08.017

[B51] Wang Y. Feinstein S. I. Fisher A. B. (2008). Peroxiredoxin 6 as an antioxidant enzyme: protection of lung alveolar epithelial type II cells from H_2_O_2_-induced oxidative stress. *J. Cell Biochem.* 104 1274–1285. 10.1002/jcb.21703 18260127 PMC4922305

[B52] Wankun X. Wenzhen Y. Min Z. Weiyan Z. Huan C. Wei D. (2011). Protective effect of paeoniflorin against oxidative stress in human retinal pigment epithelium in vitro. *Mol. Vis.* 17 3512–3522. 22219646 PMC3249435

[B53] Wu Y. Feinstein S. I. Manevich Y. Chowdhury I. Pak J. H. Kazi A. (2009). Mitogen-activated protein kinase-mediated phosphorylation of peroxiredoxin 6 regulates its phospholipase A(2) activity. *Biochem. J.* 419 669–679. 10.1042/BJ20082061 19140803 PMC2770719

[B54] Yan F. Hui Y. N. Li Y. J. Guo C. M. Meng H. (2007). Epidermal growth factor receptor in cultured human retinal pigment epithelial cells. *Ophthalmologica* 221 244–250. 10.1159/000101926 17579290

[B55] Yang D. Jin M. Bai C. Zhou J. Shen Y. (2017). Peroxiredoxin 6 suppresses Muc5ac overproduction in LPS-induced airway inflammation through H2O2-EGFR-MAPK signaling pathway. *Respir. Physiol. Neurobiol.* 236 84–90. 10.1016/j.resp.2016.11.012 27884794

[B56] Zha X. Wu G. Zhao X. Zhou L. Zhang H. Li J. (2015). PRDX6 protects ARPE-19 cells from oxidative damage via PI3K/AKT signaling. *Cell Physiol. Biochem.* 36 2217–2228. 10.1159/000430186 26279427

[B57] Zhang C. Jin Y. Marchetti M. Lewis M. R. Hammouda O. T. Edgar B. A. (2022). EGFR signaling activates intestinal stem cells by promoting mitochondrial biogenesis and β-oxidation. *Curr. Biol.* 32 3704–3719.e7. 10.1016/j.cub.2022.07.003 35896119 PMC10117080

[B58] Zhang L. Wang F. Jiang Y. Xu S. Lu F. Wang W. (2013). Migration of retinal pigment epithelial cells is EGFR/PI3K/AKT dependent. *Front. Biosci.* 2013:661–671. 10.2741/s398 23277077

[B59] Zhang T. Du W. (2015). Groucho restricts rhomboid expression and couples EGFR activation with R8 selection during Drosophila photoreceptor differentiation. *Dev. Biol.* 407 246–255. 10.1016/j.ydbio.2015.09.011 26417727 PMC4663172

[B60] Zheng Q. Ren Y. Tzekov R. Zhang Y. Chen B. Hou J. (2012). Differential proteomics and functional research following gene therapy in a mouse model of Leber congenital amaurosis. *PLoS One* 7:e44855. 10.1371/journal.pone.0044855 22953002 PMC3432120

[B61] Zhu C. Dong Y. Liu H. Ren H. Cui Z. (2017). Hesperetin protects against H_2_O_2_-triggered oxidative damage via upregulation of the Keap1-Nrf2/HO-1 signal pathway in ARPE-19 cells. *Biomed. Pharmacother.* 88 124–133. 10.1016/j.biopha.2016.11.089 28103505

[B62] Zhu Y. Zhao K. K. Tong Y. Zhou Y. L. Wang Y. X. Zhao P. Q. (2016). Exogenous NAD(+) decreases oxidative stress and protects H_2_O_2_-treated RPE cells against necrotic death through the up-regulation of autophagy. *Sci. Rep.* 6:26322. 10.1038/srep26322 27240523 PMC4886526

