# Supplementary material for: Hydrogen peroxide-induced oxidative damage and protective role of peroxiredoxin 6 protein via EGFR/ERK signaling pathway in RPE cells
Source: Front Aging Neurosci. 2023 Jul 17;15:1169211. doi: 10.3389/fnagi.2023.1169211 (PMC10388243; doi:10.3389/fnagi.2023.1169211)
Supplement: Supplementary file 1 [file Data_Sheet_1.PDF]

FIG. 1

Fig 1A

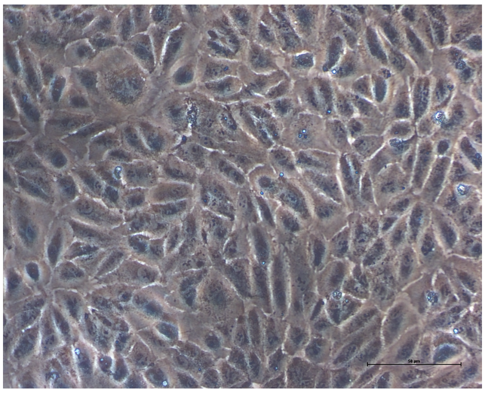

control

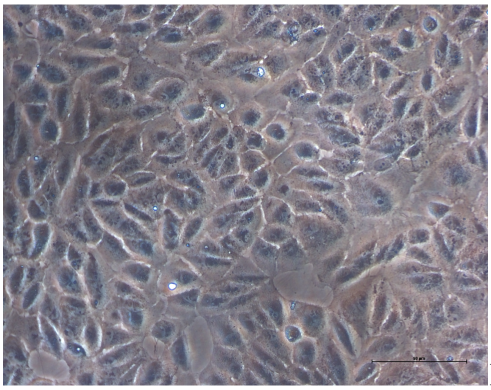

H_2O_2 100uM

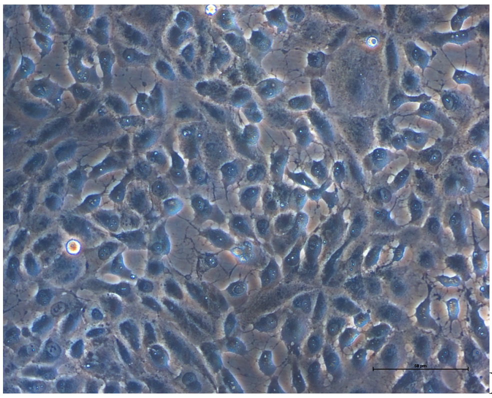

H_2O_2 300uM

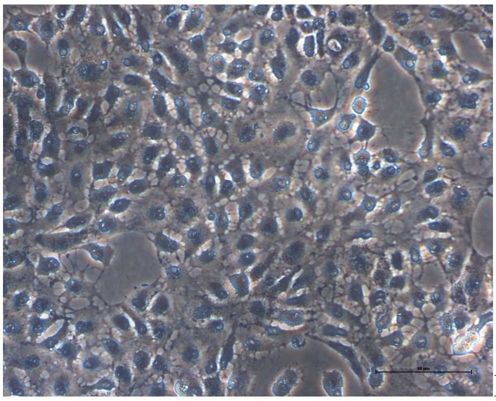

H_2O_2 500uM

Fig 1B

MTT assay data

	Control	H2O2 100	H2O2 300	H2O2 500
sample	0.995233556	1.006673022	0.770257388	0.240228789
sample	1.002859867	0.949475691	0.827454719	0.228789323
sample	0.968541468	0.930409914	0.846520496	0.224976168
sample	1.03336511	0.93422307	0.861773117	0.255481411
sample	0.959816303	0.93422307	0.872560276	0.523536165
sample	0.996555683	0.950631458	0.877152698	0.574052813
sample	0.996555683	1.024110218	0.881745121	0.555683123
sample	1.047072331	0.959816303	0.872560276	0.58783008
sample	1.056179775	0.934831461	0.916853933	0.476404494
sample	0.961797753	0.988764045	0.858426966	0.530337079
sample	0.966292135	0.939325843	0.858426966	0.498876404
sample	1.015730337	0.952808989	0.862921348	0.512359551
平均值	1	0.958774424	0.858887775	0.434046283

Fig1C

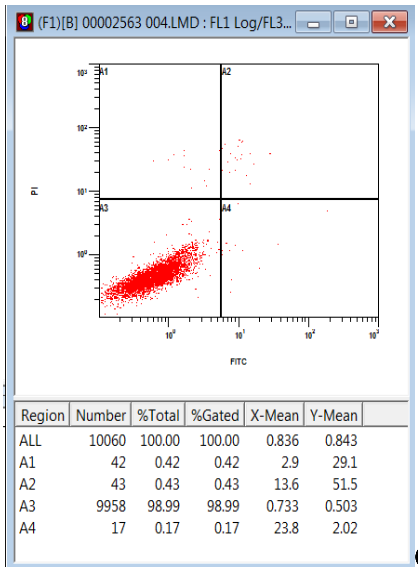

Control

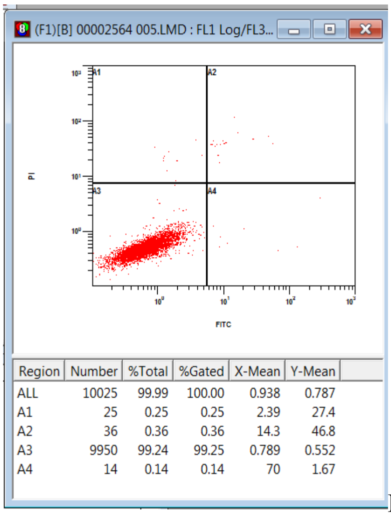

Region	Number	%Total	%Gated	X-Mean	Y-Mean
ALL	10025	99.99	100.00	0.938	0.787
A1	25	0.25	0.25	2.39	27.4
A2	36	0.36	0.36	14.3	46.8
A3	9950	99.24	99.25	0.789	0.552
A4	14	0.14	0.14	70	1.67

H_2O_2 100 Mm

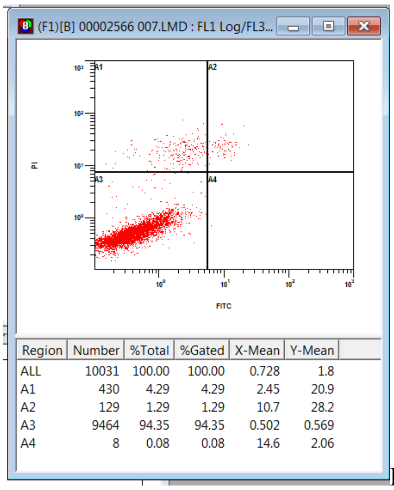

Region	Number	%Total	%Gated	X-Mean	Y-Mean
ALL	10031	100.00	100.00	0.728	1.8
A1	430	4.29	4.29	2.45	20.9
A2	129	1.29	1.29	10.7	28.2
A3	9464	94.35	94.35	0.502	0.569
A4	8	0.08	0.08	14.6	2.06

H_2O_2 300 Mm

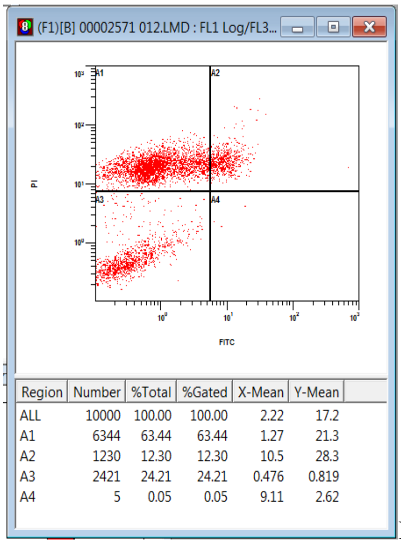

Region	Number	%Total	%Gated	X-Mean	Y-Mean
ALL	10000	100.00	100.00	2.22	17.2
A1	6344	63.44	63.44	1.27	21.3
A2	1230	12.30	12.30	10.5	28.3
A3	2421	24.21	24.21	0.476	0.819
A4	5	0.05	0.05	9.11	2.62

H_2O_2 500 Mm

Fig1D

Cell death	control	H_2O_2 100uM	H_2O_2 300uM	H_2O_2 500uM
sample	1.36%	2.15%	5.66%	55.22%
sample	1.23%	1.40%	5.04%	74.63%
sample	1.42%	0.43%	4.59%	75.79%
average	1.34%	1.33%	5.10%	68.55%

Fig1E

Control

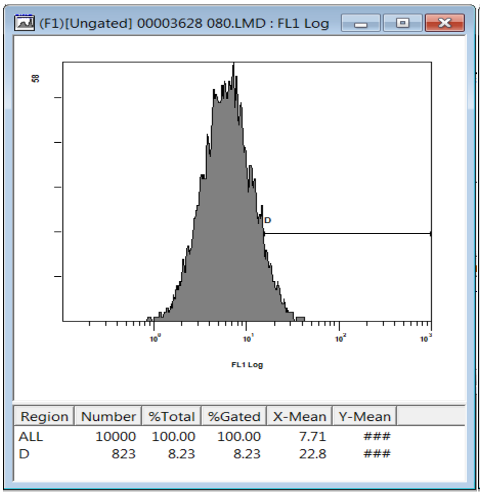

H_2O_2 100

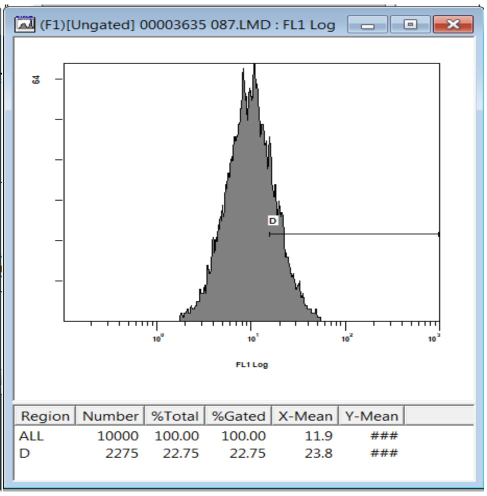

H_2O_2 300

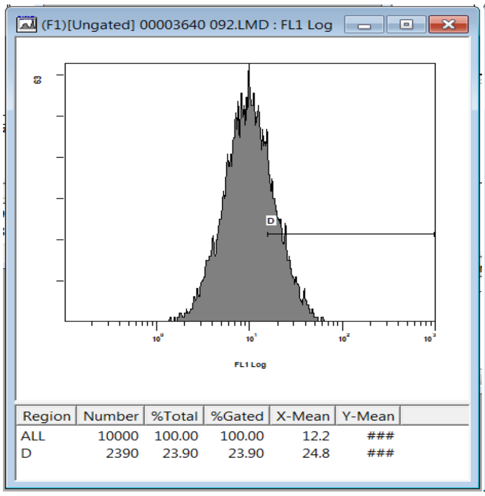

H_2O_2 500

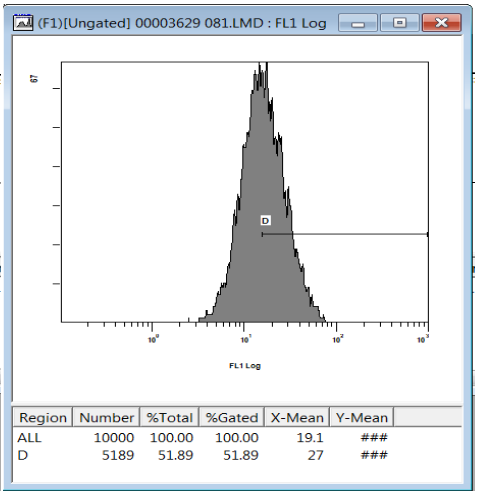

Fig1F

	ct	H_2O_2 100	H_2O_2 300	H_2O_2 500
sample	7.71	11.9	15	19.1
sample	6.01	10.7	12.2	17
sample	5.56	11.4	11.9	15.5
average	6.4	11.3	13.0	17.2
ratio	1	1.76	2.03	2.68
