# Supplementary material for: Hydrogen peroxide-induced oxidative damage and protective role of peroxiredoxin 6 protein via EGFR/ERK signaling pathway in RPE cells
Source: Front Aging Neurosci. 2023 Jul 17;15:1169211. doi: 10.3389/fnagi.2023.1169211 (PMC10388243; doi:10.3389/fnagi.2023.1169211)

FIG. 2

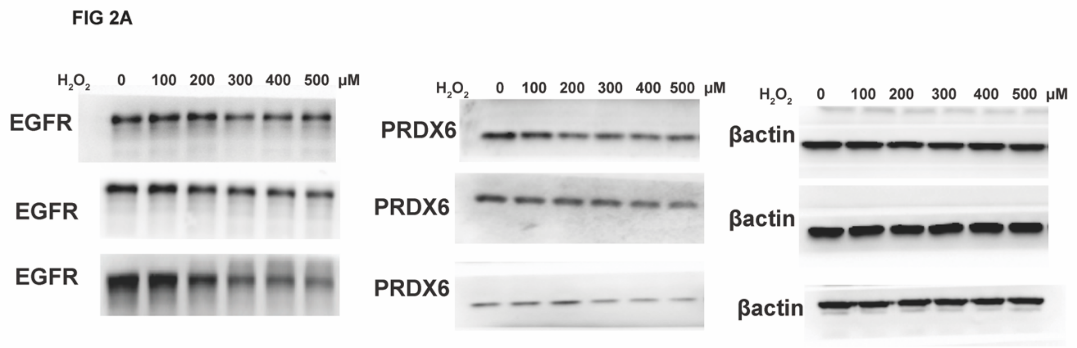

FIG 2B

EGFR	control	H2O2 100	H2O2 200	H2O2 300	H2O2 400	H2O2 500
sample 1	1	1.03	0.81	0.63	0.67	0.68
sample 2	1	0.87	1.10	0.76	0.66	0.57
sample 3	1	0.97	0.82	0.68	0.69	0.56
average	1	0.96	0.91	0.69	0.67	0.60

PRDX6	contro 1	H2O2 100	H2O2 200	H2O2 300	H2O2 400	H2O2 500
sample 1	1	0.76	0.44	0.50	0.46	0.53
sample 2	1	0.89	0.99	0.67	0.58	0.42
sample 3	1	0.77	0.58	0.16	0.18	0.19
average	1	0.81	0.67	0.44	0.41	0.39

Fig2C

Control EGFR H_2O_2 EGFR

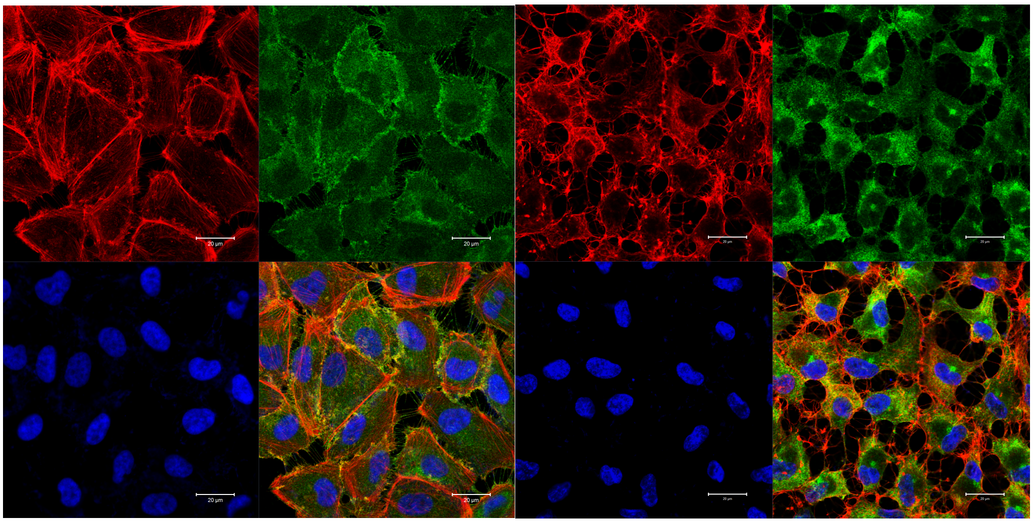

Control PRDX6 H_2O_2 PRDX6

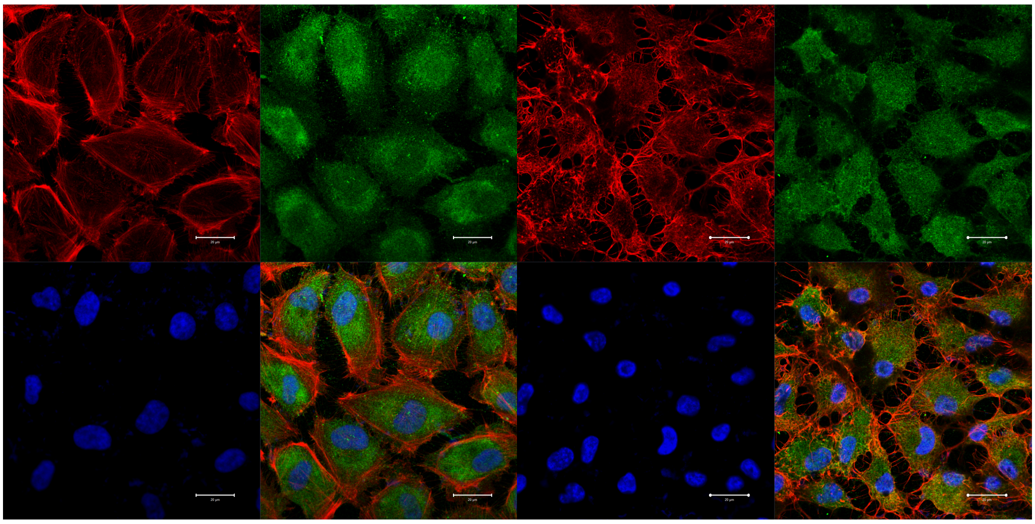

Supplement: Supplementary file 2 [file Data_Sheet_2.PDF]
