# Supplementary material for: Hydrogen peroxide-induced oxidative damage and protective role of peroxiredoxin 6 protein via EGFR/ERK signaling pathway in RPE cells
Source: Front Aging Neurosci. 2023 Jul 17;15:1169211. doi: 10.3389/fnagi.2023.1169211 (PMC10388243; doi:10.3389/fnagi.2023.1169211)
Supplement: Supplementary file 3 [file Data_Sheet_3.PDF]

FIG. 3

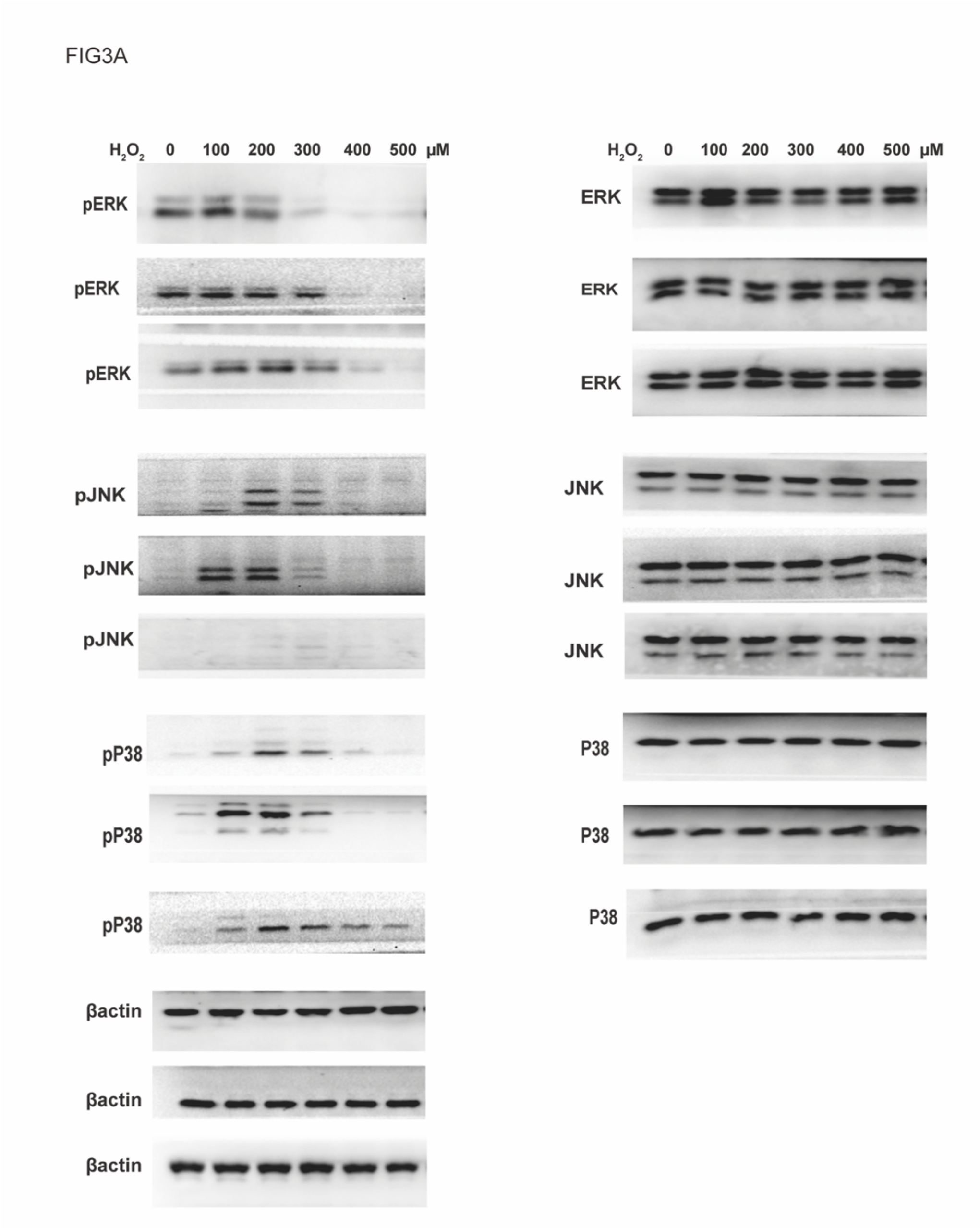

FIG. 3B

pERK	control	H2O2 100	H2O2 200	H2O2 300	H2O2 400	H2O2 500
sample 1	1	2.05	1.14	0.35	0.10	0.10
sample 2	1	1.19	0.92	0.22	0.04	0.05
sample 3	1	1.20	0.95	0.23	0.04	0.15
sample 4	1	1.17	1.05	0.89	0.15	0.10

average	1	1.40	1.01	0.42	0.08	0.10

ERK	control	H2O2 100	H2O2 200	H2O2 300	H2O2 400	H2O2 500
sample 1	1	1.2	0.97	0.85	0.93	1.05
sample 2	1	1.1	1.23	1.14	1.02	1.17
sample 3	1	1.08	1.15	1.14	1.02	1.18
sample 4	1	1.08	0.91	0.88	0.74	0.60
average	1	1.12	1.06	0.99	0.93	1.00

pP38	control	H2O2 100	H2O2 200	H2O2 300	H2O2 400	H2O2 500
sample 1	1	5.13	10.51	3.73	2.51	2.93
sample 2	1	4.79	17.13	10.51	3.09	1.05
sample 3	1	4.86	21.53	4.95	5.01	5.58
average	1	4.93	16.39	6.40	3.53	3.19

P38	control	H2O2 100	H2O2 200	H2O2 300	H2O2 400	H2O2 500
sample 1	1	1.04	0.99	1.11	1.18	1.27
sample 2	1	0.96	0.91	0.96	1.06	1.06
sample 3	1	0.90	0.88	0.95	1.03	1.13
average	1	0.97	0.93	1.01	1.09	1.15

pJNK	control	H2O2 100	H2O2 200	H2O2 300	H2O2 400	H2O2 500

sample 1	1	1.53	3.07	5.29	3.34	1.96
sample 2	1	5.14	5.04	1.98	1.02	1.29
sample 3	1	1.89	7.86	4.94	1.78	1.65
average	1	2.86	5.33	4.07	2.04	1.61

JNK	control	H2O2 100	H2O2 200	H2O2 300	H2O2 400	H2O2 500
sample 1	1	1.31	0.96	1.17	1.34	1.27
sample 2	1	1.05	1.16	1.07	1.49	1.26
sample 3	1	1.10	0.89	0.96	0.82	0.73
average	1	1.16	1.00	1.06	1.20	1.09

FIG.4A

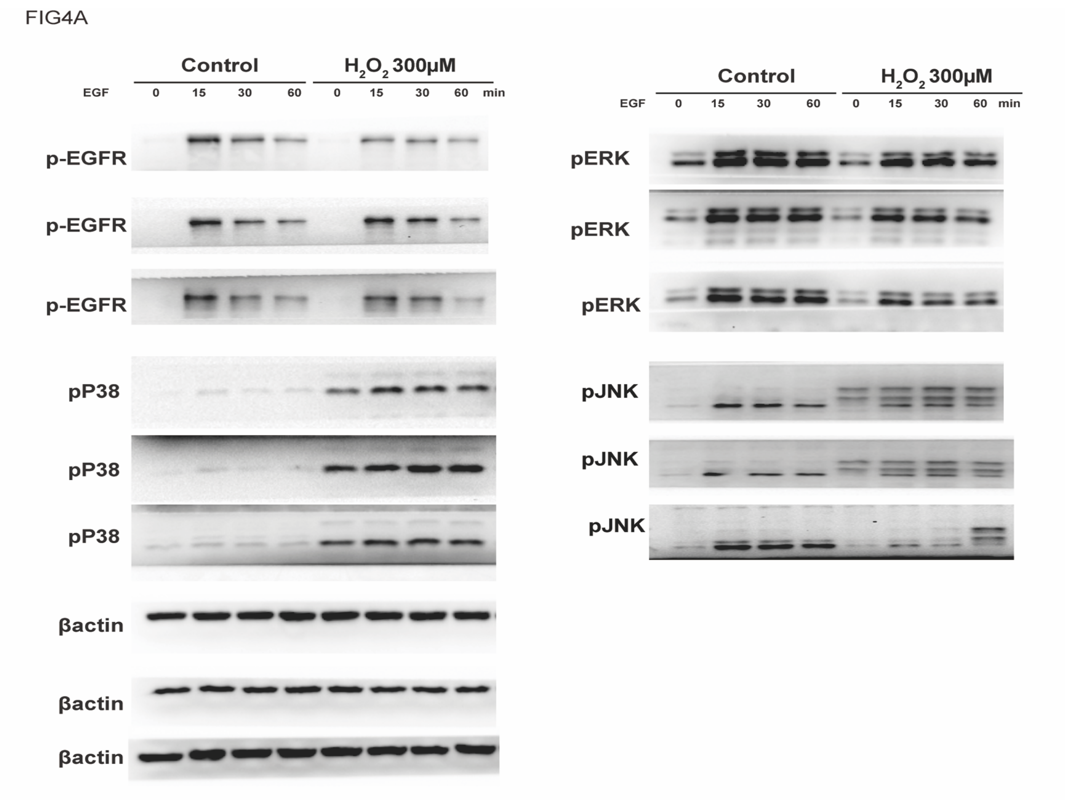

FIG.4B

EGFR

CONTROL	H_2O_2 300
EGF	EGF

0 min	15 min	30 min	60 min	0 min	15 min	30 min	60 min
1	29.1	20.5	11.43	1.02	15.95	15.15	9.78
1	19.3	11.2	7.62	1.26	16.63	12.80	4.10
1	26.57	19.05	10.61	1.76	17.20	14.22	10.35
1	24.97	16.90	9.89	1.35	16.60	14.06	8.07

pERK

CONTROL				H_2O_2 300			
EGF				EGF			
0 min	15 min	30 min	60 min	0 min	15 min	30 min	60 min
1	2.64	2.65	2.34	0.79	1.97	1.91	1.54
1	3.59	3.21	3.08	0.88	2.28	2.01	1.37
1	3.33	3.18	3.07	0.95	2.64	2.42	1.79

pPJNK

CONTROL				H_2O_2 300			
EGF				EGF			
0 min	15 min	30 min	60 min	0 min	15 min	30 min	60 min
1	5.27	5.51	5.02	7.37	9.85	10.94	9.22
1	5.00	4.97	4.49	5.41	8.08	8.54	7.12
1	3.41	3.25	2.68	4.51	6.25	6.63	5.63

pP38

CONTROL				H_2O_2 300			
EGF				EGF			
0 min	15 min	30 min	60 min	0 min	15 min	30 min	60 min
1	2.45	2.10	3.05	14.50	23.16	22.08	21.43
1	1.80	1.36	1.52	10.27	16.00	13.48	7.72
1	2.49	2.13	3.01	15.02	24.05	22.60	22.01

FIG.5A

MTT	Control	PRDX6	H_2O_2 300	PRDX6 H_2O_2 300	H_2O_2 500	PRDX6 H_2O_2 500
sample1	0.95	1.04	0.58	0.97	0.43	0.55
sample2	0.99	1.02	0.61	0.91	0.24	0.59
sample3	1.03	1.01	0.62	0.95	0.33	0.62
sample4	0.99	1.08	0.8	0.87	0.29	0.58
sample5	0.98	0.98	0.63	0.92	0.44	0.64
sample6	1.02	1.1	1.09	0.96	0.39	0.61

FIG.5 B

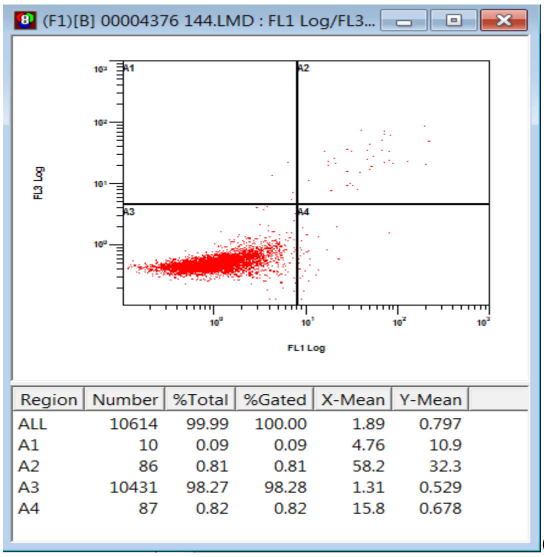

Control plasmid

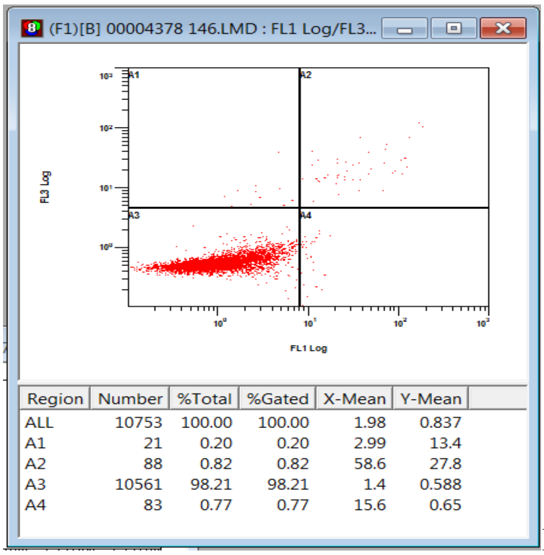

PRDX6 plasmid

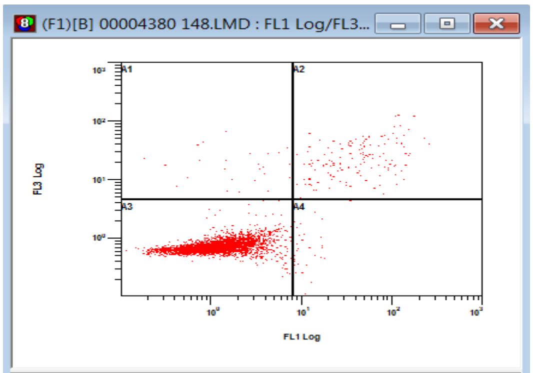

Region	Number	%Total	%Gated	X-Mean	Y-Mean
ALL	10069	100.00	100.00	2.95	1.65
A1	59	0.59	0.59	2.9	21.3
A2	278	2.76	2.76	55.2	29.3
A3	9647	95.81	95.81	1.33	0.743
A4	85	0.84	0.84	17	0.78

Control plasmid+ H_2O_2 300 uM

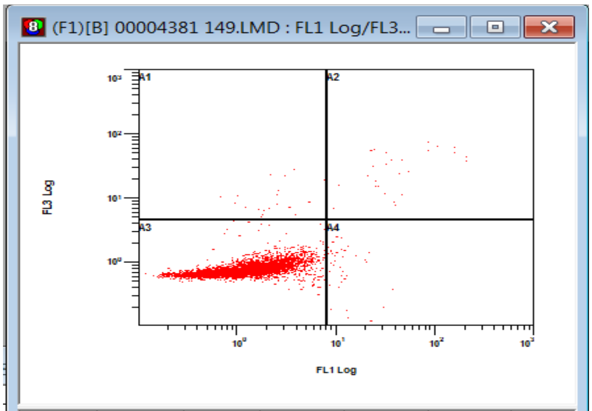

Region	Number	%Total	%Gated	X-Mean	Y-Mean
ALL	10367	100.00	100.00	1.97	1.01
A1	41	0.40	0.40	2.4	9.55
A2	60	0.58	0.58	59.7	32.5
A3	10170	98.10	98.10	1.48	0.793
A4	96	0.93	0.93	17.2	0.903

PRDX6 plasmid+ H_2O_2 300 uM

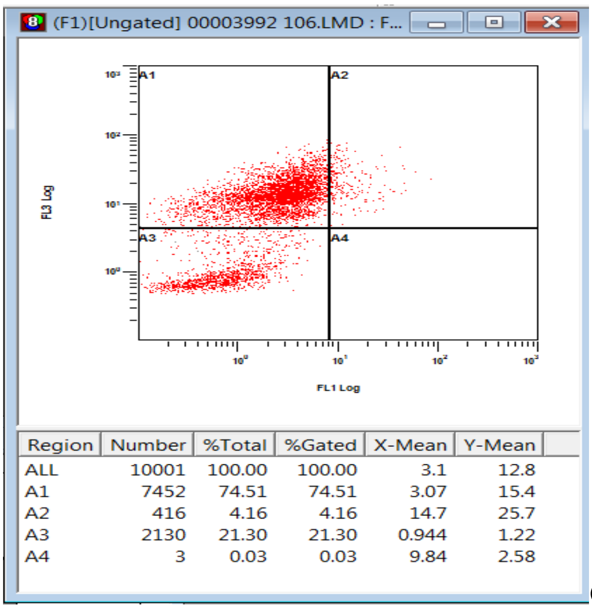

Control plasmid+ H_2O_2 500 uM

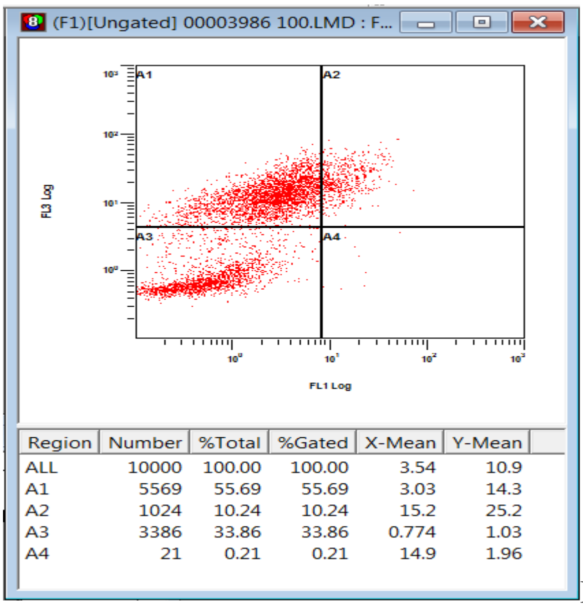

PRDX6 plasmid+ H_2O_2 500 uM

FIG.5C

cell dath	Control	PRDX6	H_2O_2 300	PRDX6 H_2O_2 300	H_2O_2 500	PRDX6 H_2O_2 500
sample 1	1.72%	1.79%	5.7%	1.91%	78.38%	65.75%
sample 2	1.55%	1.96%	4.19%	1.97%	77.69%	70.42%
sample 3	1.27%	1.48%	6.69%	2.59%	76.77%	70.67%

FIG.5D

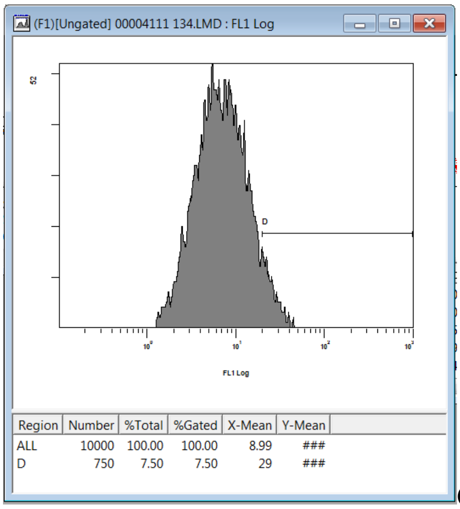

Control plasmid

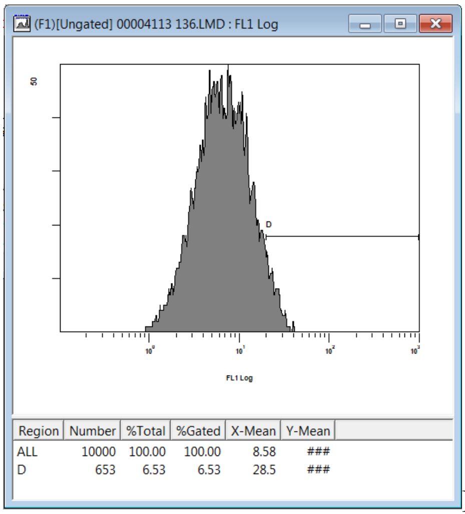

PRDX6 plasmid

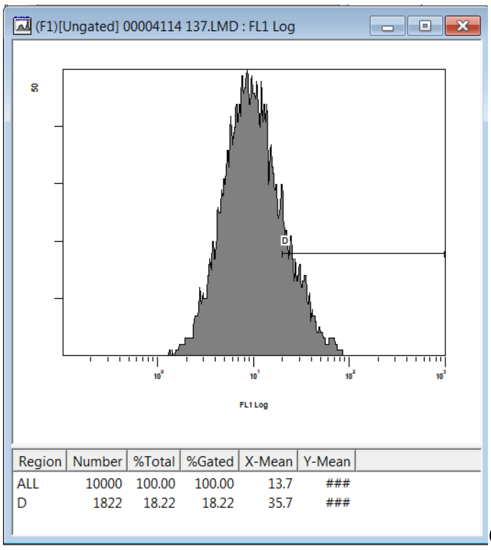

Control plasmid+ H_2O_2 300 uM

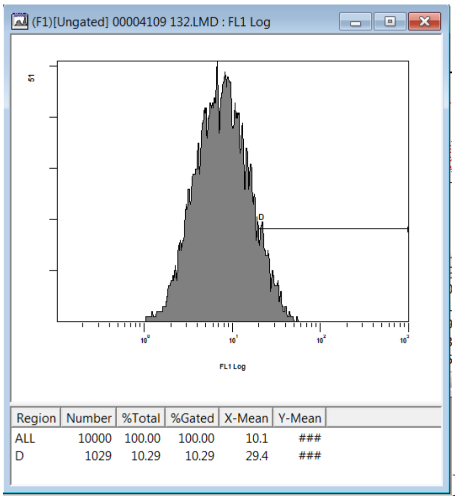

PRDX6 plasmid+ H_2O_2 300 uM

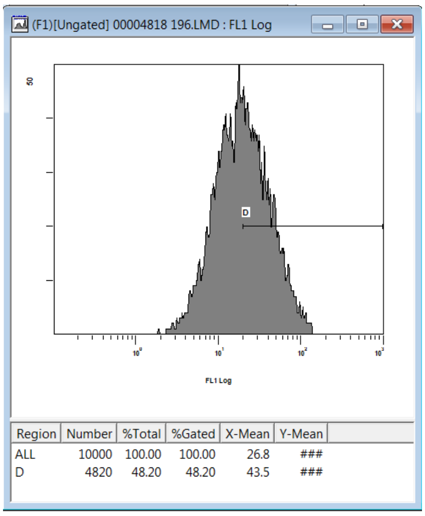

Control plasmid+ H_2O_2 500 uM

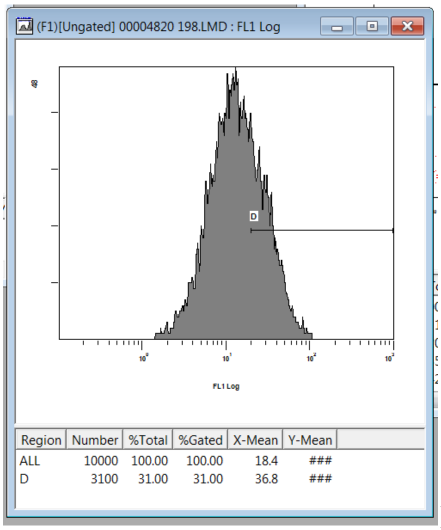

PRDX6 plasmid+ H_2O_2 500 uM

FIG.5D

ROS	Control	PRDX6	H_2O_2 300	PRDX6 H_2O_2 300	H_2O_2 500	PRDX6 H_2O_2 500
sample 1	1.02	0.98	1.57	1.03	2.69	2.05
sample 2	0.87	1.02	1.56	1.14	3.21	1.86
sample 3	1.01	0.81	1.50	1.34	3.05	2.09
