# Supplementary material for: Hydrogen peroxide-induced oxidative damage and protective role of peroxiredoxin 6 protein via EGFR/ERK signaling pathway in RPE cells
Source: Front Aging Neurosci. 2023 Jul 17;15:1169211. doi: 10.3389/fnagi.2023.1169211 (PMC10388243; doi:10.3389/fnagi.2023.1169211)
Supplement: Supplementary file 4 [file Data_Sheet_4.PDF]

FIG.6

FIG6A

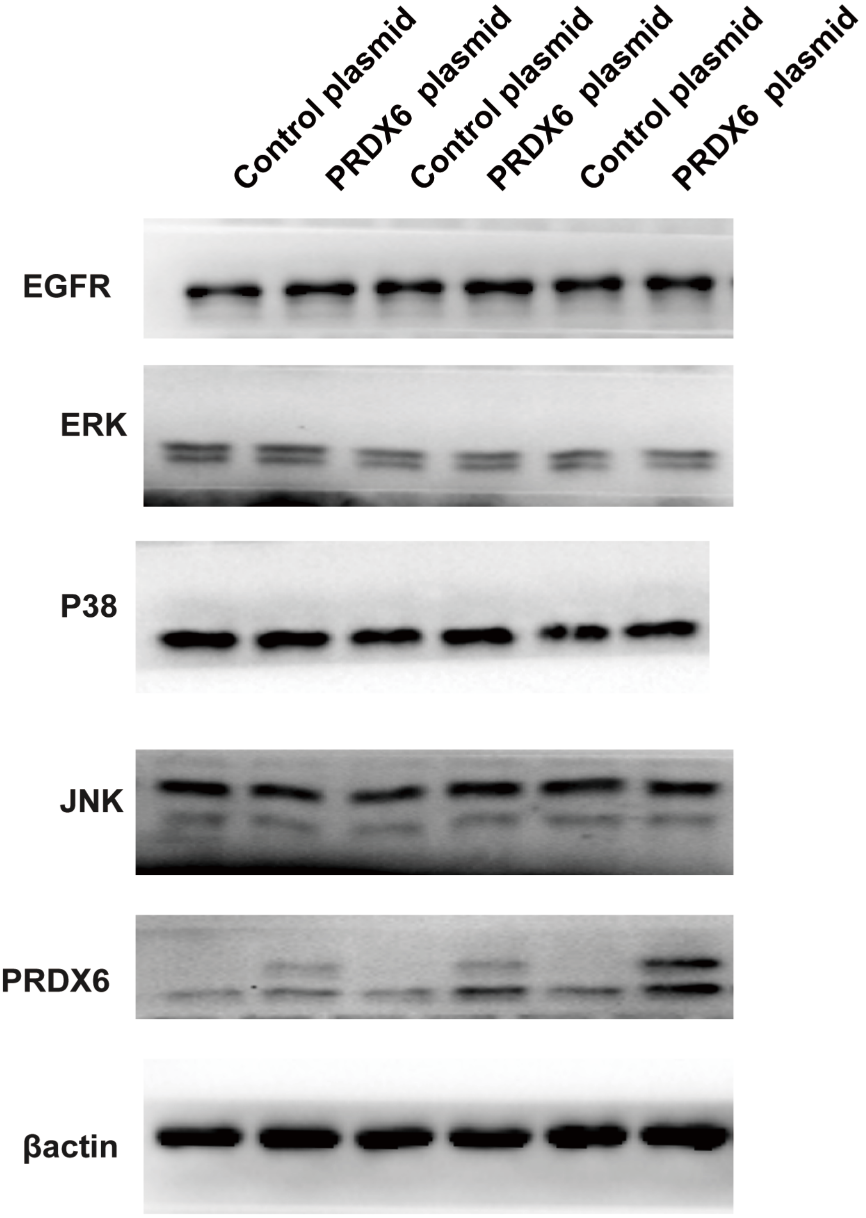

FIG.6B

control Plasmid	PRDX6 Plasmid	control Plasmid	PRDX6 Plasmid	control Plasmid	PRDX6 Plasmid	control Plasmid	PRDX6 Plasmid	control Plasmid	PRDX6 Plasmid
EGFR		ERK		P38		JNK		PRDX6	
1.00	1.35	1.00	1.50	1.00	0.95	1.00	1.22	1.00	4.15
1.00	1.23	1.00	0.87	1.00	1.11	1.00	0.79	1.00	2.79
1.00	1.14	1.00	1.12	1.00	1.18	1.00	1.29	1.00	6.05

FIG.6C

Control plasmid

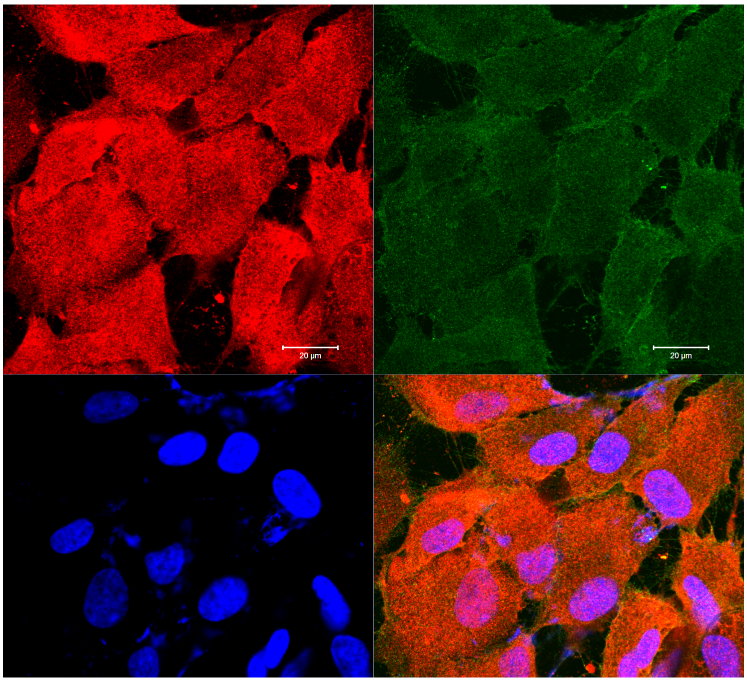

PRDX6 plasmid

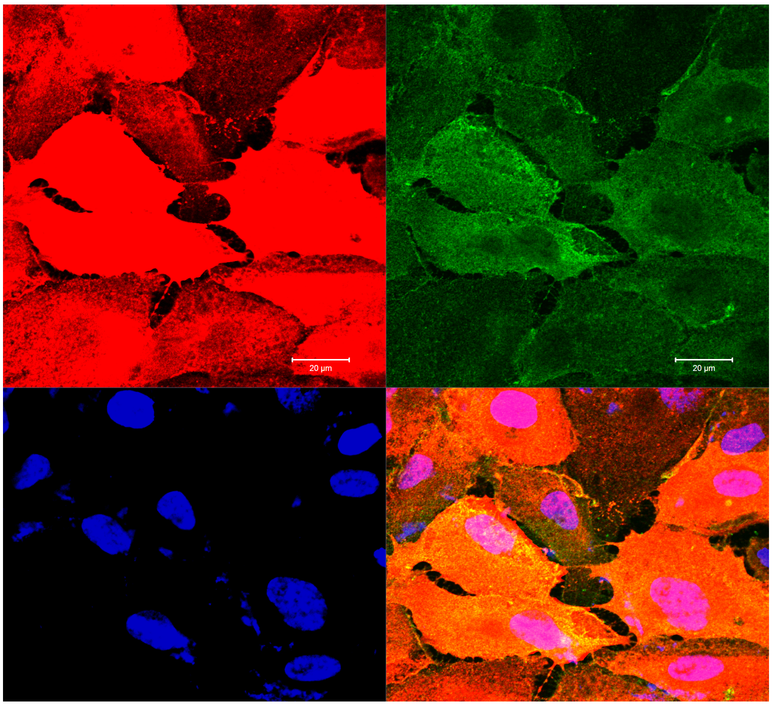

FIG.6D

Fig 6 D

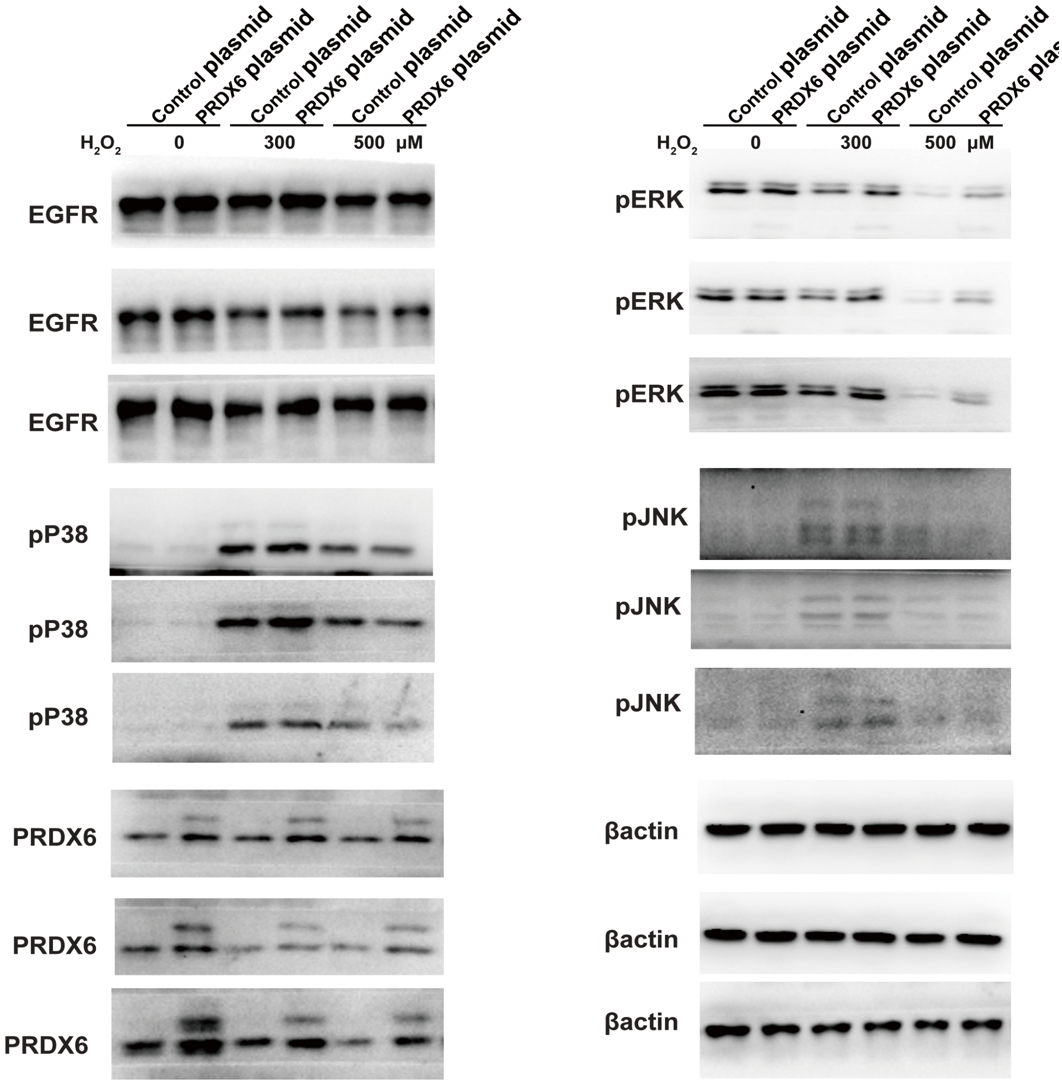

FIG.6E

EGFR

Control	PRDX6	H_2O_2 300	PRDX6 H_2O_2 300	H_2O_2 500	PRDX6 H_2O_2 500
1	1.07	0.85	0.90	0.69	0.80
1	1.04	0.92	1.01	0.72	0.76
1	1.11	0.95	0.98	0.73	0.77

pERK

Control	PRDX6	H_2O_2 300	PRDX6 H_2O_2 300	H_2O_2 500	PRDX6 H_2O_2 500
1	0.97	0.61	0.75	0.08	0.32
1	1.068	0.61	0.81	0.08	0.30
1	0.98	0.72	0.77	0.12	0.19

pP38

Control	PRDX6	H_2O_2 300	PRDX6 H_2O_2 300	H_2O_2 500	PRDX6 H_2O_2 500
1	0.85	8.87	11.75	5.99	4.57
1	1.04	10.98	10.93	4.58	3.49
1	1.23	7.39	8.38	5.03	4.16

pJNK

Control	PRDX6	H_2O_2 300	PRDX6 H_2O_2 300	H_2O_2 500	PRDX6 H_2O_2 500
1	0.99	3.37	3.29	1.43	1.72
1	1.44	2.05	2.59	1.07	1.46
1	0.93	3.29	3.31	2.31	0.95

FIG.7A

Fig. 7A

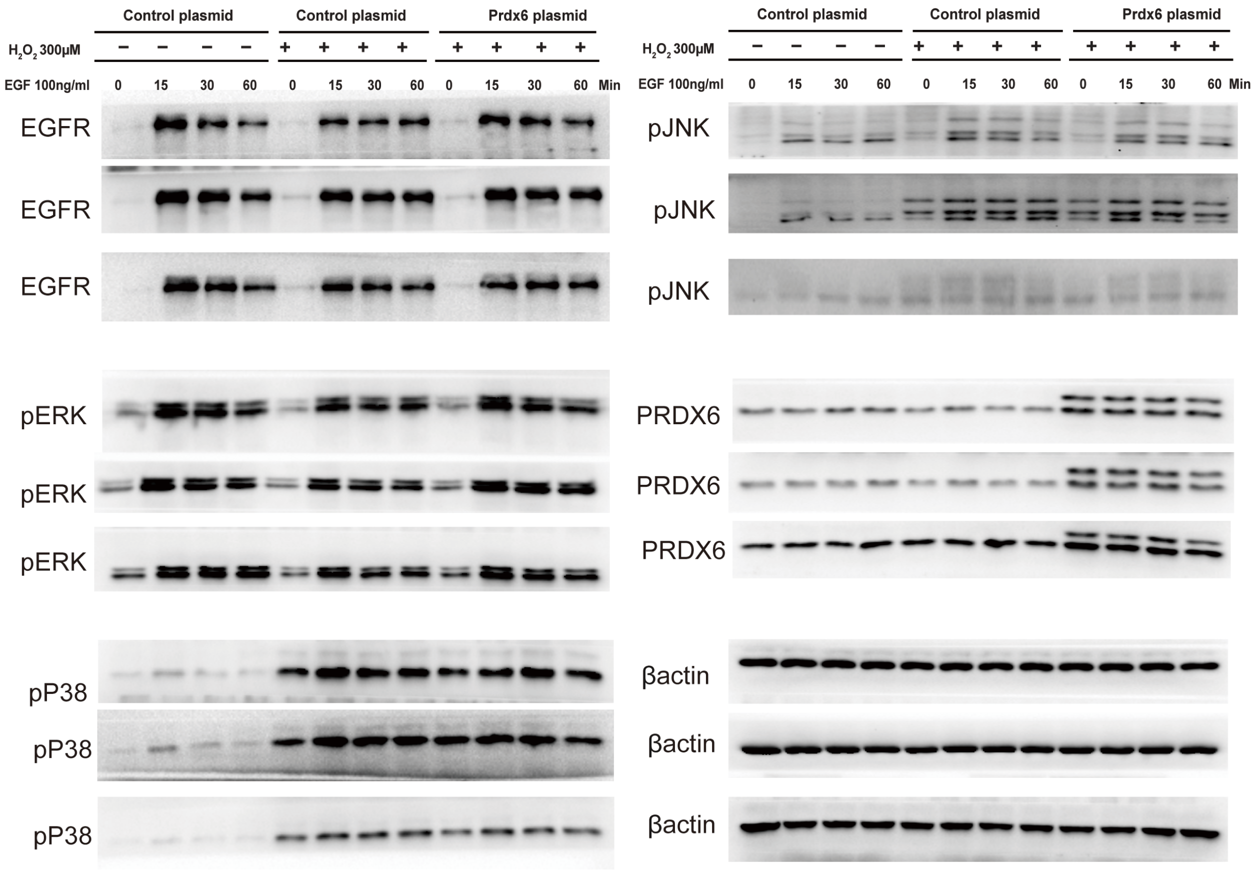

FIG.7B

pEGFR

	Control plasmid				Control plasmid				PRDX6 plasmid			
H_2O_2	-	-	-	-	+	+	+	+	+	+	+	+
EGF	0	15	30	60	0	15	30	60	0	15	30	60
	1.00	20.36	16.32	12.55	1.56	15.21	14.18	14.15	1.66	17.85	15.49	13.45
	1.00	18.67	17.53	12.93	2.15	12.19	13.60	12.63	1.70	18.39	19.29	16.11
	1.00	22.28	15.43	11.31	0.77	14.54	13.94	14.46	2.71	19.03	17.91	13.32

pERK

	Control plasmid				Control plasmid				PRDX6 plasmid			
H_2O_2	-	-	-	-	+	+	+	+	+	+	+	+
EGF	0	15	30	60	0	15	30	60	0	15	30	60
	1.00	0.93	1.26	4.06	3.10	4.12	3.46	2.77	3.79	2.72	2.49	3.32
	1.00	0.72	1.23	3.64	3.24	3.75	3.45	2.69	3.11	2.53	2.67	2.69
	1.00	0.72	1.23	3.72	3.35	3.86	3.45	2.74	3.19	2.60	2.76	2.77

pJNK

	Control plasmid				Control plasmid				PRDX6 plasmid			
H_2O_2	-	-	-	-	+	+	+	+	+	+	+	+
EGF	0	15	30	60	0	15	30	60	0	15	30	60
	1.00	3.22	2.56	1.80	3.53	6.53	6.14	6.56	4.70	6.88	5.98	4.01
	1.00	1.92	1.88	2.42	2.27	3.31	3.12	2.41	2.00	2.75	2.39	1.78
	1.00	2.64	2.55	3.66	3.20	5.15	4.79	3.66	2.71	4.20	3.45	2.38

pP38

	Control plasmid				Control plasmid				PRDX6 plasmid			
H_2O_2	-	-	-	-	+	+	+	+	+	+	+	+
EGF	0	15	30	60	0	15	30	60	0	15	30	60
	1.00	3.51	1.67	1.64	13.83	23.26	20.07	18.84	13.44	16.98	20.22	15.81
	1.00	4.04	3.56	3.57	13.21	21.11	18.68	18.44	18.01	19.20	18.77	13.92
	1.00	2.11	1.89	3.16	18.77	25.21	23.74	25.42	16.44	23.68	19.61	19.03
