# Supplementary figures and images for: Hydrogen peroxide-induced oxidative damage and protective role of peroxiredoxin 6 protein via EGFR/ERK signaling pathway in RPE cells
Source: Front Aging Neurosci. 2023 Jul 17;15:1169211. doi: 10.3389/fnagi.2023.1169211 (PMC10388243; doi:10.3389/fnagi.2023.1169211)

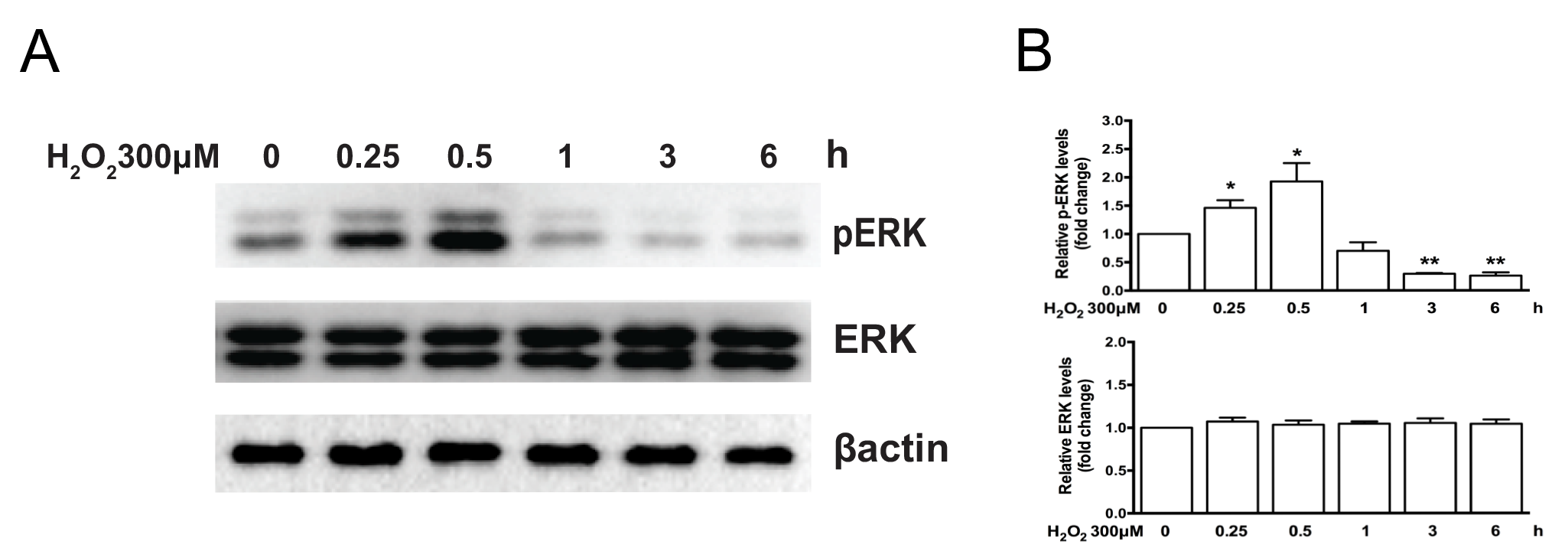

Supplement: Supplementary Figure 1 — Treatment with 300 μM H2O2 for different times affects pERK in ARPE-19 cells. (A) Western blot results of pERK and ERK after ARPE-19 cells were treated with 300 μM H2O2 for 0, 0.25, 0.5, 1, 3 or 6 h. (B) Quantitative analysis of western blot results from three independent experiments. *P < 0.05, **P < 0.01, compared to control. [file Image_1.TIF]
